# The relationship between visual acuity loss and GABAergic inhibition in amblyopia

**DOI:** 10.1162/imag_a_00256

**Published:** 2024-08-06

**Authors:** I. Betina Ip, William T. Clarke, Abigail Wyllie, Kathleen Tracey, Jacek Matuszewski, Saad Jbabdi, Lucy Starling, Sophie Templer, Hanna Willis, Laura Breach, Andrew J. Parker, Holly Bridge

**Affiliations:** Wellcome Centre for Integrative Neuroimaging, FMRIB Building, Nuffield Department of Clinical Neurosciences, University of Oxford, John Radcliffe Hospital, Oxford, United Kingdom; Laboratory of Brain Imaging, Nencki Institute of Experimental Biology, Polish Academy of Sciences, Warsaw, Poland; Orthoptics Department, Oxford Eye Hospital, John Radcliffe Hospital, Oxford, United Kingdom; Department of Physiology, Anatomy and Genetics, University of Oxford, Oxford, United Kingdom; Institut für Biologie, Otto-Von-Guericke Universität, Magdeburg, Germany

**Keywords:** amblyopia, GABAergic inhibition, visual cortex, MRS, visual acuity

## Abstract

Early childhood experience alters visual development, a process exemplified by amblyopia, a common neurodevelopmental condition resulting in cortically reduced vision in one eye. Visual deficits in amblyopia may be a consequence of abnormal suppressive interactions in the primary visual cortex by inhibitory neurotransmitter γ-aminobutyric acid (GABA). We examined the relationship between visual acuity loss and GABA+ in adult human participants with amblyopia. Single-voxel proton magnetic resonance spectroscopy (MRS) data were collected from the early visual cortex (EVC) and posterior cingulate cortex (control region) of 28 male and female adults with current or past amblyopia while they viewed flashing checkerboards monocularly, binocularly, or while they had their eyes closed. First, we compared GABA+ concentrations between conditions to evaluate suppressive binocular interactions. Then, we correlated the degree of visual acuity loss with GABA+ levels to test whether GABAergic inhibition could explain visual acuity deficits. Visual cortex GABA+ was not modulated by viewing condition, and we found weak evidence for a negative correlation between visual acuity deficits and GABA+. These findings suggest that reduced vision in one eye due to amblyopia is not strongly linked to GABAergic inhibition in the visual cortex. We advanced our understanding of early experience dependent plasticity in the human brain by testing the association between visual acuity deficits and visual cortex GABA in amblyopes of the most common subtypes. Our study shows that the relationship was not as clear as expected and provides avenues for future investigation.

## 1 Introduction

Amblyopia is a neurodevelopmental visual disorder associated with lifelong loss of normal spatial vision. At ~3%, amblyopia remains the most common visual impairment in children and adults (Birch, 2013; Fu et al., 2020) across geographical boundaries (Hu et al., 2022; Pan et al., 2009). Amblyopia has been investigated in numerous species, including cats (Hubel & Wiesel, 1965; Wiesel & Hubel, 1963), non-human primates (Hallum et al., 2017; Kiorpes, 2019), and humans (Barnes et al., 2001; Clavagnier et al., 2015; Joly & Franko, 2014), reviewed by (Mitchell & Sengpiel, 2018). While these studies shed light on the structural and functional abnormalities in the early and secondary visual cortex, no single neural correlate appears to account for the severity and the diversity of deficits observed in amblyopia.

It has long been thought that intracortical inhibition plays a role in amblyopia (Burchfield & Duffy, 1981; Sengpiel & Blakemore, 1996). The strongest evidence in support of this theory comes from animal models. Notably, iontophoretic application of GABA_a_ antagonist bicuculline diminished intracortical suppression in V1 of amblyopic (Burchfield & Duffy, 1981) and strabismic cats (Sengpiel et al., 2006). Amblyopic eye responses in adult rats were also rescued by chronic infusion of anti-depressant fluoxetine and abolished by GABA_a_ agonist diazepam (Maya Vetencourt et al., 2008). Finally, a single dose of ketamine rapidly reduced parvalbumin interneuron driven inhibition, rescuing vision in the amblyopic eye of adult mice (Grieco et al., 2020). These findings raise the possibility of pharmacologically treating amblyopia beyond the critical period, a period in early development where experience can alter brain function (Hensch & Quinlan, 2018). However, attempts to replicate some of the effects from interventions developed in animals in human amblyopes have produced mixed results (Huttunen et al., 2018; Lagas et al., 2019; Sharif et al., 2019). Thus, preclinical findings from animals with less developed visual systems may not directly translate to the complex visual system of primates (Mitchell & Sengpiel, 2018).

A handful of studies support a relationship between visual cortex GABA and eye dominance in normally sighted people using binocular rivalry, a psychophysical proxy of cortical inhibition. While these neuroimaging studies have reported different behavioral metrics, they all reported a link to visual cortical GABA levels (Ip et al., 2021; Lunghi et al., 2015; Pitchaimuthu et al., 2017; Robertson et al., 2016; van Loon et al., 2013). A similar neural mechanism may underlie pathological eye dominance in the amblyopic visual system. Only a single study to our knowledge has tested this possibility; however, the study was limited to a small number of participants who were either anisometropic or mixed amblyopes (Mukerji et al., 2022), and did not include strabismic amblyopes. It is well known that amblyopia is linked to various causes, the two main ones being strabismus, misalignment of the optical axes, or differences in optic blur between eyes (anisometropia). These risk factors may have different effects on the visual system.

Our study tested the link between GABAergic inhibition and visual acuity deficits in 28 participants with amblyopia, including amblyopia of anisometropic, strabismic and mixed etiologies. We presented visual stimuli inside the MRI scanner and measured GABA+ in the early visual cortex, expecting varying levels of visual suppression to be revealed depending on monocular or binocular visual stimulation. Contrary to our hypothesis, we found no mean differences in GABA+ across different viewing conditions. When we averaged across conditions and correlated visual acuity deficits with GABA+, we found weak evidence for a negative relationship suggesting the greater the visual acuity loss, the lower GABA+ levels in the early visual cortex. An exploration of the association between visual acuity deficits and GABA+ by subtype suggested that the relationship can be influenced by amblyopic etiology.

## 2 Methods

### 2.1 Participants

Twenty-eight adult amblyopic participants (14 females, age, *M* = 30, *SD* = 8 years) with a history or presence of unilateral amblyopia and with no other ocular pathology or neurological condition took part in the MRI study. Participants were identified from the general population by self-reported history of amblyopia, eye-patching and/or corrective surgery for strabismus, and/or a strongly dominant eye and were then diagnosed with current or past unilateral amblyopia by a research orthoptist prior to the MRI scan. Current amblyopia was formally diagnosed using the criterion of >= 0.2 logarithm of the minimum angle of resolution (LogMAR) difference in visual acuity (VA) between the amblyopic and fellow eye. Three out of 28 participants were former amblyopes, that is, ex-amblyopes, who were treated with occlusion therapy in childhood and had <0.2 LogMAR difference in visual acuity between eyes. Because their difference in visual acuity was less than the diagnostic criterion for amblyopia, they are referred to as ‘ex-amblyopes’. The ex-amblyopes were included to represent participants who experienced abnormal binocular vision in childhood and whose amblyopia was successfully treated. Participants were representative of different aetiologies of amblyopia: strabismic (n = 13) amblyopes had a history of previous strabismus with or without surgery, anisometropes (n = 10) had more optic blur in one eye, and mixed (n = 5) amblyopes experienced both strabismus and optic blur. For one participant (sub-017), the data involving visual stimulation (MRS, fMRI) could not be used due to a visual display error, although the resting data (‘eyes closed’) acquisition was collected successfully. On the day of the MRI scan, participants were instructed to avoid consuming caffeine. A payment of £40 was made for the 2 h MRI session. All volunteers gave informed and written consent, as approved by the University of Oxford Research Ethics Committee (Ethics Approval Reference: R75202/RE002, ‘Neurochemistry and the amblyopic brain’). Exclusion criteria were previous neurological or psychiatric abnormality, orthoptic abnormality other than lazy eye, pregnancy or breast-feeding, frequent cigarette use (more than 1 cigarette per day in the past 3 months), alcohol consumption (more than 14 units of alcohol/week, over 3 days or more), and migraine with aura.

#### 2.1.1 Sample size rationale

We had no prior MR spectroscopy data from amblyopic participants to use in an a priori sample size calculation, thus our sample size was based on resource constraints (Lakens, 2022). Using G*Power for a post-hoc sensitivity analysis, we calculated that a Pearson’s correlation coefficient with 28 participants would be sensitive to a minimum of *r* = 0.32 with 80% power (alpha = 0.05, one-tailed). This means that our dataset would not be able to reliably detect correlations smaller than *r* = 0.32. For the exploratory subtype analysis, participants were separated into three groups according to their type of amblyopia (anisometropic, strabismic, mixed anisometropic and strabismic). Our study was not designed to evaluate sub-type specific effects, hence these subgroups were statistically underpowered and at risk of effect inflation (Button et al., 2013).

### 2.2 Clinical measures

Participants underwent full orthoptic screening with visual correction, if any, at the Orthoptic Department, John Radcliffe Hospital, Oxford, UK. No further refractive correction was provided as part of the study. Outcome measures from the orthoptic report were the presence or absence of current amblyopia and the type of amblyopia. Monocular visual acuity using Snellen or EDTRS visual acuity was converted to LogMAR units by considering the additional letters that were read or missed (Tiew et al., 2020). As a supporting measure we also report pinhole-corrected visual acuity (PCVA). In cases where a pinhole measure was performed on the amblyopic eye but not the fellow eye, the non-pinhole fellow eye VA was used to calculate the difference in pinhole visual acuity (phΔVA). The orthoptic screening results are shown in Table 1. The absolute difference in visual acuity of the fellow eye minus the amblyopic eye (abs(FE-AE) = ΔVisual Acuity) was the correlate for MRI measures.

**Table 1.** Orthoptic assessment of amblyopic participants.

Sub	Sex	Age	Occ	Years	Type	Rx	FE VA LogMAR	AE VA LogMAR	ΔVA LogMAR	phΔVALogMAR	Stereo arcsec
Sub-001	M	36	Yes	~7	Strab	RE: -0.5; LE: -1	-0.18	0.6	0.78	0.78	600
Sub-002	M	34	No	~32	Aniso	N/A	-0.12	0.78	0.9	0.9	600
Sub-003	M	37	Yes	4-5	Strab	RE: +3.00; LE: +3.75	-0.06	0.52	0.58	0.58	N/A
Sub-004	F	41	Yes	3-4	Aniso	RE: +3.25; LE: N/A	-0.04	0.8	0.84	0.64	-ve
Sub-005	F	36	Yes	4	Aniso	RE: -1; LE: +3	-0.18	0.1	0.28	0.28	85
Sub-006*	M	28	Yes	8-9	Strab	RE: -8; LE: -8.5	-0.06	0	0.06	0.02	85
Sub-007	F	24	Yes	6-7	Aniso	N/A	-0.16	1.04	1.2	1	600
Sub-008*	F	20	Yes	4-5	Strab	RE: -0.25; LE: plano	-0.06	-0.04	0.02	0.02	85
Sub-009*	M	19	Yes	~6-7	Mixed	RE: +6; LE: +7.5	-0.14	0	0.14	0.14	N/A
Sub-010	M	20	Yes	4-5	Strab	N/A	-0.02	0.48	0.5	0.32	-ve
Sub-011	F	24	Yes	4	Aniso	RE: +0.5; LE: +5	0.02	1	0.98	0.46	N/A
Sub-012	M	40	Yes	~3	Strab	RE: -1.75; LE: -1.75	0.18	1	0.82	0.82	-ve
Sub-013	F	43	Yes	3-4	Mixed	RE: 0; LE: +3.5	-0.14	0.18	0.32	0.32	300
Sub-014	M	22	Yes	4	Mixed	RE: +2.5; LE: plano	-0.08	0.16	0.24	0.24	N/A
Sub-015	M	38	Yes	5	Aniso	N/A	-0.14	0.6	0.74	0.74	N/A
Sub-016	F	22	Yes	4	Aniso	RE: +3.25; LE: +1.25	0	0.36	0.36	0.32	110
Sub-017	F	25	No	7-8	Aniso	RE: plano; LE: -0.5	-0.06	1	1.06	0.66	215
Sub-018	M	27	Yes	4	Strab	N/A	-0.16	0.78	0.94	0.94	-ve
Sub-019	M	27	Yes	8	Aniso	RE: +1; LE: +3.5	-0.14	0.18	0.32	0.32	85
Sub-020	M	22	Yes	3-4	Mixed	RE: +2.25; LE: +3.25	-0.16	0.78	0.94	0.94	N/A
Sub-021	F	28	Yes	5	Strab	RE: +6; LE: +6	0	0.78	0.78	0.78	N/A
Sub-022	M	38	Yes	6	Strab	N/A	0.02	1.1	1.08	1.08	N/A
Sub-023	F	37	Yes	3	Mixed	RE: -1.25; LE: -6.00	-0.08	1.22	1.3	1.3	N/A
Sub-024	F	19	Yes	4	Strab	RE: +3; LE: +3	0.02	0.3	0.28	0.26	300
Sub-025	F	32	Yes	5-6	Aniso	RE: +1.13; LE: +4.38	-0.18	0.8	0.98	0.98	600
Sub-026	M	35	Yes	2-3	Strab	N/A	-0.18	1	1.18	1.18	N/A
Sub-027	F	25	Yes	2-8	Strab	RE: -0.5; LE: 0	0.14	0.56	0.42	0.56	-ve
Sub-028	F	40	No	11	Strab	N/A	-0.08	0.22	0.3	0.3	N/A

Sub = subject; F = female, M = male; Occ = occlusion therapy; Years = age detected; Aniso = anisometropic amblyopia, Strab = strabismic amblyopia, Mixed = mixed anisometropic and strabismic amblyopia; AE = amblyopic eye; FE = fellow eye; Rx = visual correction in dioptres; RE = right eye; LE = left eye; plano = balance lens; FE VA = fellow eye visual acuity in LogMAR; AE VA = amblyopic eye visual acuity in LogMAR; ΔVA = visual acuity difference between amblyopic and fellow eye in LogMAR; phΔVA = visual acuity difference using pinhole measure; Stereo = threshold on Frisby Stereopsis Test in arcsec; -ve = negative; N/A = not acquired. * = Former amblyopes with visual acuity loss < 0.2 LogMAR.

### 2.3 Magnetic resonance imaging

Magnetic resonance images from all participants were collected using a 3T Siemens Prisma (Siemens Healthineers AG, Erlangen, Germany), equipped with a 64-channel head and neck coil. A 1-mm isotropic whole-head T1-weighted anatomical image (MPRAGE, TR = 2000 ms; TE = 2.03 ms; field-of-view= 256 x 256 mm; 208 slices; flip angle = 8°) was collected for registration purposes with a total acquisition time of 5 min 31 s. A 2 mm isotropic multiband gradient echo sequence was used for the fMRI-localizer experiment (MB4; TR = 1355 ms; TE = 32.4 ms; field-of-view = 192 x 192; 72 slices; flip angle = 70°). In total, 144 volumes were collected, with a total scan duration of 3 min 20 s. MEGA-PRESS data (Mescher et al., 1998) was acquired with a locally developed version of the sequence, derived from the CMRR spectroscopy package MEGA-PRESS sequence. Acquisition parameters were as follows: MRS-voxel size: anterior to posterior = 20 mm, left to right = 25 mm, head to foot = 25 mm; echo time (TE) = 68 ms, repetition time (TR) = 1500 ms; 160 edit-on and 160 edit-off spectra per condition; VAPOR and dual-band editing pulse water suppression; 22.3 ms editing pulse using a 53 Hz bandwidth, which was centered at 1.9 ppm (edit-on) and at 7.5 ppm (edit-off) in alternation; 16-step phase cycling; and 8 min 13 s run time per condition. For the EVC voxel placement, the region of interest was first centered to the occipital midline to cover equivalent portions of the right and left visual cortex, then angled to be parallel to the calcarine sulcus, and moved as posterior as possible while avoiding contamination by the cerebellar tentorium and the sagittal sinus. A control voxel was positioned at the midline in the posterior cingulate cortex (PCC). The PCC is a suitable control location because it is non-overlapping with the occipital lobe, and it has been used at 7T using non-edited sequences (Lunghi et al., 2015) and at 3T with edited sequences (Rideaux, 2020) as a control voxel for data from the early visual cortex. The PCC voxel data were acquired with identical acquisition parameters, voxel size, and angle as the EVC voxel.

#### 2.3.1 Stimulation paradigm inside the MRI scanner

**Fig. 1.**
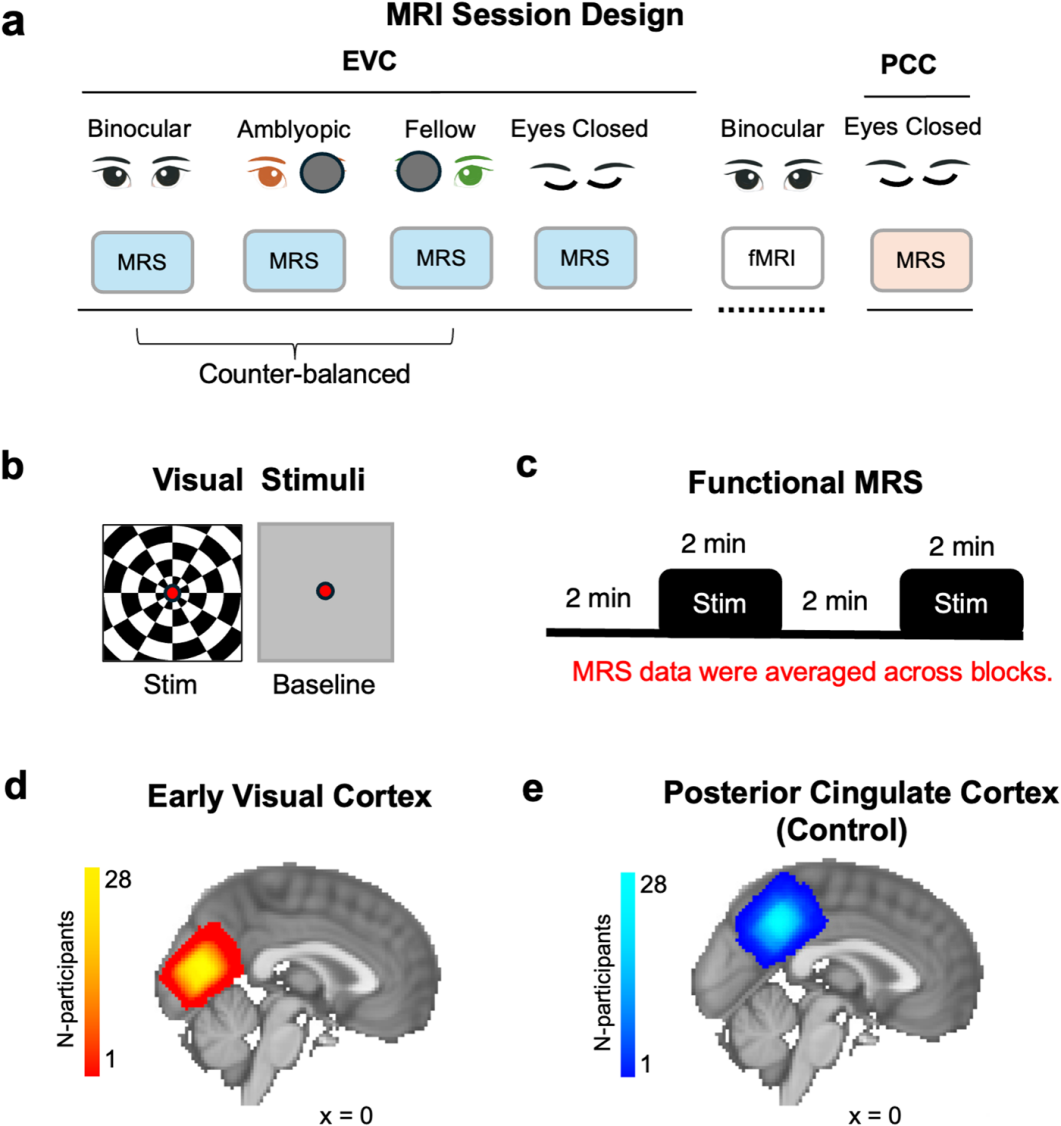
Diagram of the experimental design and the group MRS voxel positions. (a) The MRI session started with three runs where participants viewed visual stimuli with both eyes, or with the amblyopic or fellow eye while MRS data were measured in the early visual cortex (EVC). After the visual stimulation runs, data were acquired from the EVC while participants had their eyes closed. This was followed by a short fMRI localizer, presenting flashing checkerboards. Finally, MRS data were acquired from the posterior cingulate cortex (PCC) while participants had their eyes closed. (b) Visual stimulation consisted of 100% contrast checkerboard stimuli, contrast reversing at 8 Hz. The baseline consisted of a blank, mid-grey screen. A fixation dot was always present, and a simple fixation task was performed throughout each visual stimulation run. (c) Each functional MRS run consisted of two alternations of 128 s grey screen followed by 128 s flashing checkerboards. MRS data were averaged across stimulus and baseline blocks. (d) The EVC voxel was placed in the bilateral posterior occipital cortex, as shown by the MRS group voxel (heat map) composed of the summed voxel position across participants. (e) The control voxel (blue map) was placed in the bilateral posterior cingulate cortex. The group MRS voxels were displayed on a sagittal slice (x = 0 mm) of the MNI-152 2 mm standard brain template.

Visual stimuli were displayed using Matlab (v.2021b) and PsychToolbox-3 (v.3.0.11). Stimuli were presented using an MR-compatible gamma-linearized LCD screen (BOLDscreen 32, Cambridge Research Systems, Cambridge, UK) positioned at the back of the 3T scanner bore. The screen had a pixel resolution of 1920 x 1200, an aspect ratio of 8:5, and a refresh rate of 60 Hz. The screen was positioned at a viewing distance of 127.5 cm. Participants viewed stimuli presented at the back of the bore through a first-silvered mirror that was fixed to the head coil at a 45° angle. The study extended a previously developed functional MRS paradigm at 7T (Ip et al., 2021; Lunghi et al., 2015), designed to test if stimuli seen through either the strong eye, the weak eye or seen binocularly could reveal differences in GABAergic inhibition during visual processing in the early visual cortex. An eyes closed ‘rest’ condition was collected as a baseline to contrast with general effects of visual stimulation (Kurcyus et al., 2018). The MRI session consisted of four early visual cortex (EVC) MRS runs: three visual stimulation runs (fellow eye, FE, amblyopic eye, AE, binocular, BE) counterbalanced for order effects, followed by an ‘eyes closed’ scan (Fig. 1a). During monocular conditions, a black eye patch occluded the non-viewing eye (Clavagnier et al., 2015). Visual stimulation consisted of full-field flashing checkerboards, contrast reversing at 8 Hz with a white fixation dot in the center and a mid-grey baseline (Fig. 1b). Each block was 128 s in duration, with baseline and stimulation blocks alternating twice in each run (Fig. 1c). A central fixation task was performed throughout the experimental run to stabilize eye position and control for attentional allocation. Participants’ MRI-safe prescription goggles were matched to their spherical refractive corrections from the orthoptic screening as closely as possible. Prior to scanning, strabismic and mixed amblyopes underwent a prism cover test at a viewing distance of stimuli displayed inside the MRI scanner (127.5 cm) to correct for squint. Glass prisms were used to re-align the fixation position of the deviating amblyopic eye during binocular viewing and removed during monocular viewing. An fMRI localizer scan (16 s stimulus - 16 s baseline, 6 cycles) was collected with the same visual stimulus and under binocular viewing after the MRS scans, to confirm the overlap of the EVC voxel (Fig. 1d) with visual regions and the non-overlap with the voxel placed in the posterior cingulate cortex control region (Fig. 1e, PCC). The corrective prism was not used during the fMRI localizer scan.

#### 2.3.2 MRS analysis

MRS data were analyzed using FSL-MRS v.2.1.19 (Clarke et al., 2021), part of the open-source FSL toolbox. First, MRS data were converted from TWIX to NIfTI format using spec2nii v.0.7.4 (Clarke et al., 2022). Then, data were pre-processed using fsl_mrs_preproc_edit for edited MRS data. It included the following steps: coil-combination, windowed averaging of phase and frequency alignment between repeats, eddy current correction, truncation of the FID to remove three time-domain points before the echo center, removal of residual water peak using Hankel Lanczos singular value decomposition (HLSVD) over 4.5–4.8 ppm, and phase and frequency alignment between averaged edit-on and edit-off spectra using spectral registration on the 2.5 to 3.5 ppm range. The processing also outputs a phase corrected non-water suppressed reference acquired immediately before the water suppressed data. The model fitting of the SVS data was implemented using a Linear Combination model as described in (Clarke et al., 2021). In essence, basis spectra were fitted to the complex-valued spectrum in the frequency domain by scaling, shifting, and broadening them. Basis spectra were grouped into two metabolite groups, with macromolecular peaks allowed to broaden and shift independently of other metabolites. The model fitting was achieved using the truncated Newton algorithm as implemented in Scipy. A complex polynomial baseline was also concurrently fitted (order = 0). To model metabolites in the edit-on minus edit-off difference spectrum, we used a simulated basis set containing the model spectra for N-acetylaspartate (NAA), N-acetylaspartateglutamate (NAAG), γ-amino-butyric acid (GABA), glutamine (Gln), glutamate (Glu), glutathione (GSH), macromolecules (MM), and combined NAA+NAAG, Glu+Gln+GSH, GABA+sysMM, with internal reference limits between 1.8–2.2 ppm (https://git.fmrib.ox.ac.uk/wclarke/win-mrs-basis-sets). Because the GABA signal at 3.0 ppm contains co-edited macromolecule signals, as well as homocarnosine (Rothman et al., 1997), the signal is referred to as GABA+ macromolecules (GABA+). Metabolite units are reported in absolute concentration in millimole per kilogram (mMol/kg), corrected for tissue fraction and tissue relaxation. For a control analysis, GABA+ relative to unsuppressed water (GABA+/water) and GABA+ relative to Creatine+ Phosphocreatine (GABA+/tCr) were reported to evaluate the influence of metabolite ratio on the association between variables. GABA+/tCr was calculated by dividing the raw GABA+ values from the difference spectrum by the raw tCr values from the edit-off spectrum. The MRS voxel positions were reconstructed using FSL FAST called within FSL-MRS. FAST divides the high-resolution anatomical image into white matter, grey matter, and cerebrospinal fluid, and calculates tissue fractions within EVC and PCC voxels. The Minimum Reporting Standards Checklist for MRS is reported in the Supplementary Materials.

#### 2.3.3 MRS spectral quality

The quality of the MRS shim was measured with the full-width-half-maximum (FWHM) of the inverted NAA singlet in the difference spectrum, where broader linewidth indicates poorer shim quality (Zollner et al., 2021). The NAA signal-to-noise ratio was obtained from the edit-off spectrum by obtaining the ratio of the peak height of the NAA basis function over the standard deviation of a pure noise region after applying a matched filter to both (Clarke et al., 2021). We compared MRS quality measures of the mean early visual cortex data across conditions (EVC) and the posterior cingulate cortex (PCC), see Table 2. The comparison revealed narrower linewidth in the PCC compared to the EVC (*t*(25) = 7.34, *p* < 0.001). This suggests that shimming was better for the PCC compared to the EVC voxel. However, SNR was higher in the EVC than the PCC (Wilcoxon signed-rank test, *Z* = 2.47, *p* = 0.013), suggesting that more signal was available in the EVC voxel. Pearson’s correlations were computed to assess the relationship between MRS quality measures and GABA+ concentrations pooled across voxel locations. These showed that neither FWHM (*r* = -0.162, *p* = 0.241, *BF*_10_ = 0.33) nor SNR (*r* = 0.12, *p* = 0.38, *BF*_10_ = 0.246) correlated with GABA+. Overall, our results show that MRS spectral quality differed between voxel locations, and that the interindividual variability across voxels in these measures did not correlate with GABA+.

**Table 2.** Metrics for MRS spectral quality.

Conditions	Shim quality NAA line width (Hz)	Signal-to-noise NAA SNR
Both eyes (n = 27)	5.5 ± 0.6	155.0 ± 30.5
Fellow eye (n = 27)	5.5 ± 0.4	152.5 ± 29.1
Amblyopic eye (n = 25)	5.6 ± 0.6	154.6 ± 28.6
Rest (n = 28)	5.6 ± 0.5	150.3 ± 28.7
Mean EVC (n = 28)	5.6 ± 0.4	152 ± 27.7
PCC rest (n = 26)	4.7 ± 0.6	136.4 ± 19.2
EVC vs. PCC *p* value	<0.001	0.013

NAA = N-acetylaspartate; Hz = Hertz; SNR = signal-to-noise; EVC = early visual cortex; PCC = posterior cingulate cortex.

#### 2.3.4 Functional MRI analysis

Functional MRI data analysis used FEAT (FMRI Expert Analysis Tool) v.6.00, part of the FSL software distribution (FMRIB’s Software Library, www.fmrib.ox.ac.uk/fsl). Pre-processing was done using motion correction MCFLIRT (Jenkinson et al., 2002); non-brain tissue extraction (Smith, 2002); spatial smoothing using Gaussian kernel of FWHM = 5 mm, grand-mean intensity normalization, and high-pass temporal filtering using a cut-off of 48 s. Registration of functional images to the 1 mm isotropic T1-weighted structural image used boundary-based registration (BBR) in FLIRT (Jenkinson et al., 2002; Jenkinson & Smith, 2001). The group activation to visual stimuli was quantified using FLAME stage 1 (FMRIB’s Local Analysis of Mixed Effects). Z (Gaussianized T/F) statistic images were thresholded using clusters determined by Z > 3.1 and a (corrected) cluster significance threshold of *p* = 0.05 (Worsley, 2001). Featquery was used to measure the percentage BOLD-signal change to binocular viewing of 6 cycles of flashing checkerboards and a blank, mid-grey screen, with each block lasting 16 seconds. The BOLD signal change was measured in the bilateral EVC MRS-voxel and a bilateral probabilistic primary visual cortex mask corresponding to the region defined as Brodmann’s area 17 in 10 cyto-architectonically mapped post-mortem brains (Amunts et al., 2000). The mask was available as part of the Jülich histological atlas (Amunts et al., 2020) in the FSLeyes viewer application. The overall size of the V1 mask was thresholded to 50% to represent regions where there was reasonable overlap between participants.

#### 2.3.5 Eye movement recording inside the MRI scanner

We monitored monocular eye position using an MR-compatible eye tracker (EyeLink 1000, SR Research Limited, Ontario, Canada) during MRS scans. Eye-tracking calibration procedures were performed prior to each functional MRS condition, and eye-tracking was performed except when equipment failure or failure to get a clear view of the eye due to the visual correction frames or head position inside the head coil prevented eye-tracking. During monocular scans, the viewing eye was monitored. During binocular scans, the amblyopic eye was monitored when possible, and if not, tracking was attempted for the fellow eye. When eye-tracking was not possible, fixation stability was monitored by experimenters through the EyeLink interface. A trained researcher ensured that all participants maintained good fixation and kept their eyes open during visual stimulation runs.

### 2.4 Statistical analysis

#### 2.4.1 Statistical packages

Data analysis and visualisation scripts were written in Python (v.3.8.13) (McKinney, 2010) using the Jupyter Notebook user interface. Data analysis was performed using the pandas software library (v.2.0.3), and pingouin (v.0.5.3) (Vallat, 2018).

#### 2.4.2 Outlier analysis

The Inter Quartile Range (IQR) was calculated for GABA+ and the glutamate and glutamine complex (Glx) to identify univariate outliers that lie outside of the middle 50% range of the data distribution. ±1.5 IQR was used as the threshold of exclusion in our study which is equivalent to ±2.7 standard deviations. The number of data points excluded were EVC GABA+: amblyopic: 1; EVC Glx: both eyes: *2*; fellow: 1. PCC GABA+: 2. MRS data from participants would have been excluded if more than one EVC condition was labeled as an outlier in the GABA+ analysis, but this did not occur in any case.

#### 2.4.3 Statistical analyses

Data analysis was performed using RStudio (RStudio Version 2023.06.0 + 421). We applied a linear mixed model (lme4) to estimate the fixed effects of ‘viewing condition’ (‘AE’, ‘FE’, ‘Both’, ’Closed’) and ‘type’ (‘strabismic’, ‘anisometropic’, ‘mixed’) while including ‘sex’ and ‘age’ as fixed effect variables to test for differences in the main outcome variables with sex and age, and while controlling for participant ID as random effects to account for repeated measures within observers. We used the anova function to obtain *p* values. The interaction term was dropped when no significant interactions between fixed effects were observed. The full model syntax with interaction term was lmer(GABA+ ~ viewing condition * type * sex * age + (1|participant), data = df). The full model syntax without interaction term was lmer(GABA+ ~ viewing condition + type + sex + age + (1|participant), data = df). We used the Type II Analysis of Variance Table with Kenward-Roger’s method when no interactions were present. Type III was used when interactions were present.

Pearson’s linear correlations were used for all correlation analyses (pingouin.corr) to evaluate the linear association between two variables and to obtain the Bayes Factor. Bayes Factors give the strength of evidence for or against the alternative hypothesis using standard interpretations, for example a Bayes Factor of 1 gives no evidence, 1–3 provides weak evidence, 3–10 provides moderate evidence, 10–30 strong evidence, and 30–100 very strong evidence for H_1_ (Nuzzo, 2017). Two-tailed hypotheses were tested unless otherwise indicated in the text. Two-tailed Fisher’s *r*-to-*z*-transform tests (http://vassarstats.net/rdiff.html) were used to evaluate the significance of the difference in correlation coefficients. Uncorrected *p* values as well as Bonferroni-adjusted *p* values were reported for the confirmatory correlation analyses. Uncorrected *p* values were reported for exploratory analyses.

## 3 Results

### 3.1 Orthoptic assessment results

**Fig. 2.**
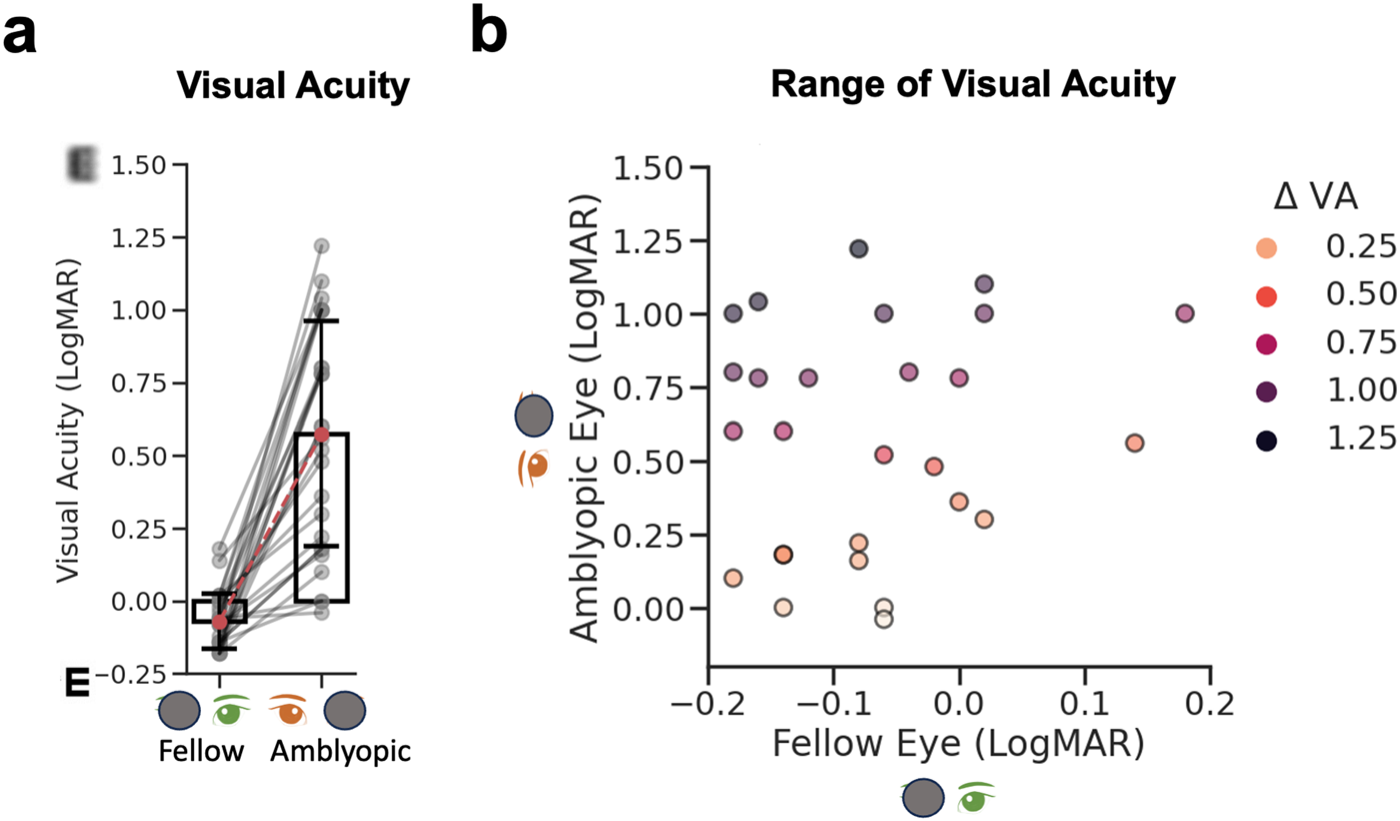
Monocular visual acuity in adult amblyopes. (a) Bar plots show the mean of the fellow eye (fellow, FE) and amblyopic eye (amblyopic, AE) visual acuity in LogMAR units. Error bars show ± 1 standard deviation. (b) AE plotted against FE visual acuity, with the color hue representing the severity of visual acuity loss (darker, more loss). Dots are individual participants. Note the different scales on the amblyopic and fellow eye axes.

The cohort consisted of 28 adult participants, including 3 with a history of monocular patching and amblyopia (Table 1). A Wilcoxon signed-rank test confirmed that the fellow eye’s visual acuity was better than the amblyopic eye (*Z* = 6.02, *p* < 0.001) (Fig. 2a). The difference in visual acuity (ΔVA), calculated by subtracting the LogMAR visual acuity of the amblyopic from the fellow eye, was used for correlation analyses with MRI measures. ΔVA ranged from 0.02 to 1.30 LogMAR (*M* = 0.66, *SD* = 0.38 LogMAR, Fig. 2b).

### 3.2 Visual cortex MRS location corresponds to visually stimulated regions

**Fig. 3.**
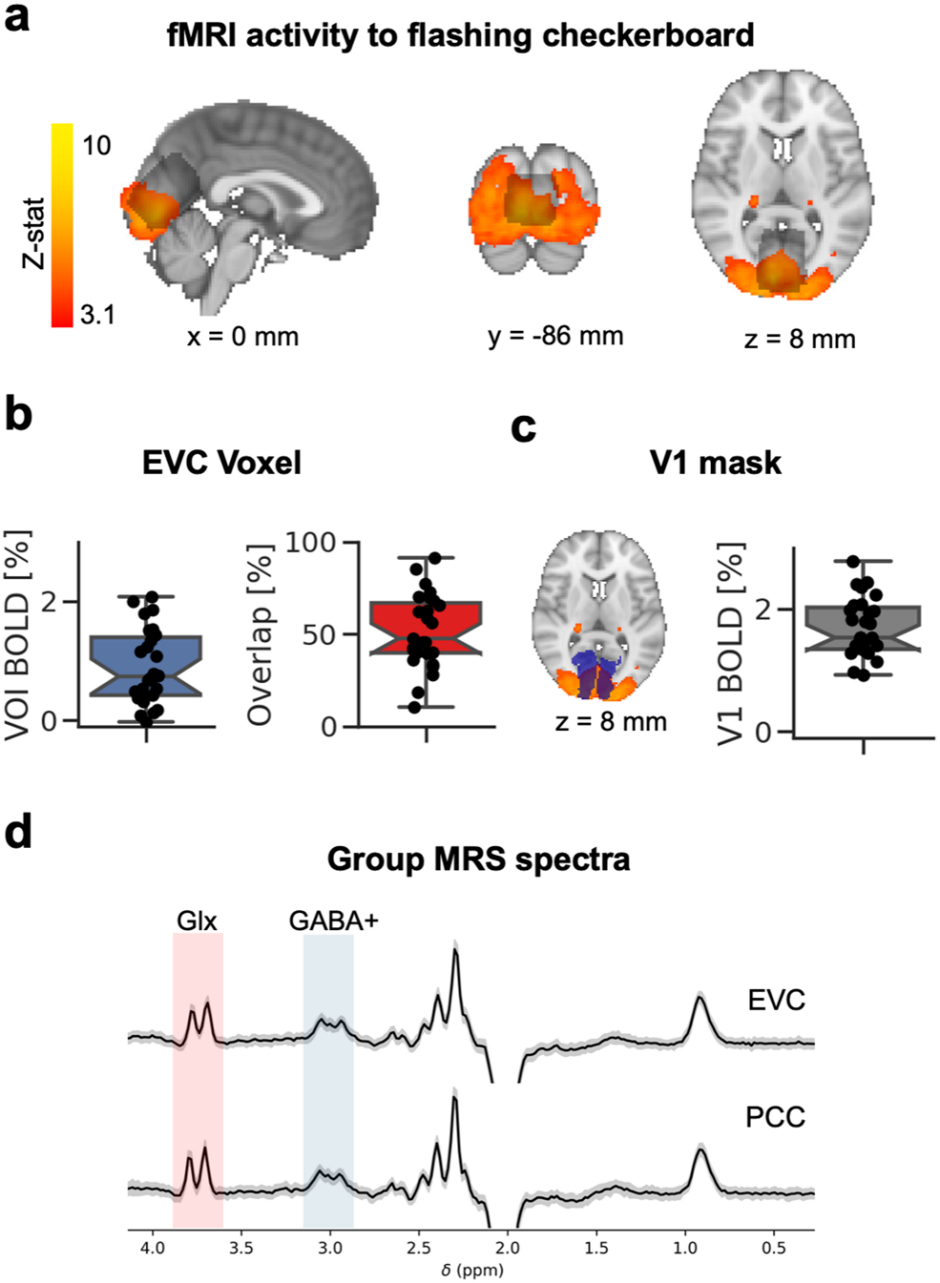
MRS validation and comparison of early visual cortex GABA+ levels across conditions. (a) Brain responses to flashing checkerboards measured with fMRI (heat map) and group early visual cortex (EVC) MRS voxel (grey map) presented on the MNI-152 2 mm standard brain (x,y,z in mm). Box-and-whisker plots show Q1 to Q3 quartile values, with a line at the median Q2 for %BOLD-signal change to flashing checkerboard compared to a blank screen inside the MRS voxel (b, left), for the percentage overlap between the MRS voxel (b, right) and the activation maps, and %BOLD-signal change inside a V1 mask (c). Whiskers show 1.5 IQR. Dots show individual participants. (d) Group EVC and PCC MRS spectra for the eyes closed rest condition (grey area = standard deviation, black line = mean) showing the most easily visually resolved signal peaks of Glx (3.75 ppm, red area) and GABA+ (3.02 ppm, blue area) peaks on the chemical shift axis. The signal is scaled to arbitrary units.

We evaluated whether the EVC MRS voxel (Fig. 3a) targeted the correct region of the cortex. Due to the MRS voxel size and avoidance of non-brain tissue, the EVC voxel could not be placed too close to the posterior edge of the brain; however, a positive BOLD signal was found within the voxel (Fig. 3b, *M* = 0.89, *SD* = 0.62 %BOLD-change). Across participants, half of the voxel’s area overlapped with the fMRI map (*M* = 51.83, *SD* = 19.92%). In a supporting analysis, we showed that the flashing checkerboard stimulus modulated the primary visual cortex activity, demonstrating the expected visual response in all participants (Fig. 3c, *M* = 1.70, *SD* = 0.48%). Across EVC and PCC voxel’s rest condition, MRS spectra were similar in appearance, with discernible GABA+ and Glx peaks (Fig. 3d).

### 3.3 No effect of viewing condition on GABA+

**Fig. 4.**
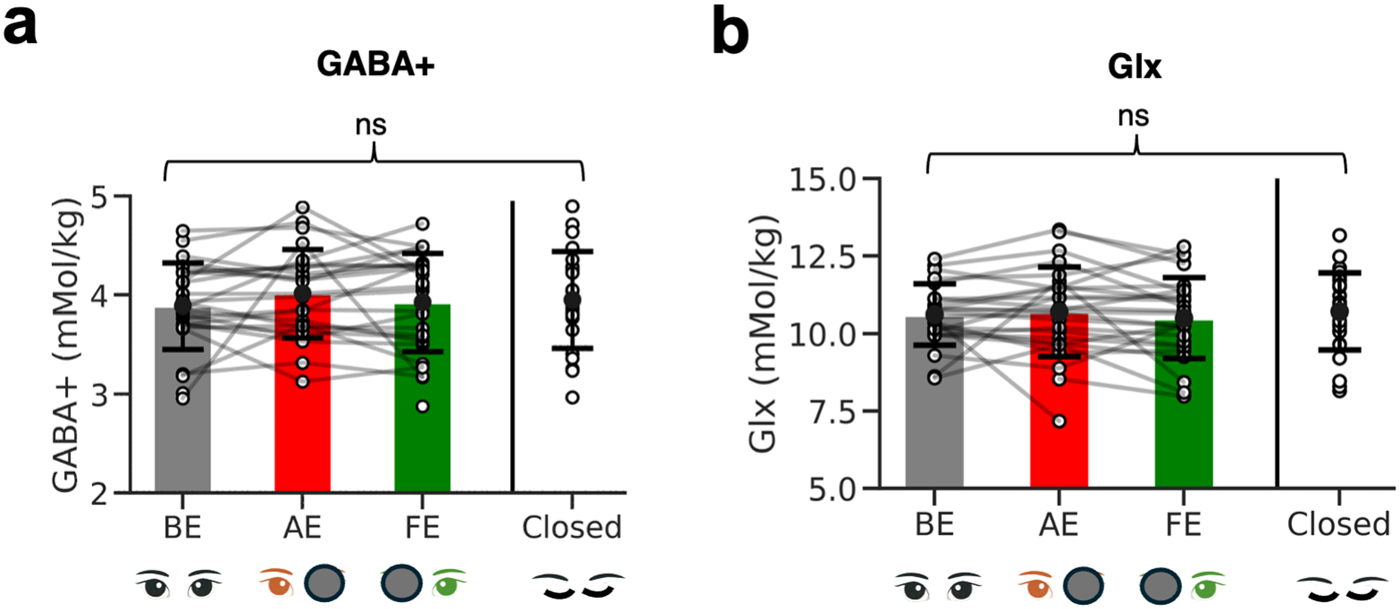
GABA+ and Glx in the visual cortex of amblyopes across conditions. (a) Bar plots show comparisons of average metabolite levels across visually stimulated conditions and the control condition (eyes closed) for early visual cortex (EVC) GABA+ and (b) Glx. Error bars show +/- 1 standard deviation. Individual dots show participants.

We tested our main prediction that viewing condition modulated visual cortex GABA+ in adult amblyopes (i.e., whether the stimulus was presented to the AE, FE, both ‘BE’, or ‘Closed’) using a linear mixed-model analysis. A significant model fit for viewing condition would have meant that the specific eye or eyes used for viewing affected the neurochemical response. However, no significant model fit was found (Fig. 4a, LMM, *F*_3,76.517_ = 0.70, *p* = 0.55). We also analyzed the glutamate + glutamine signal (Glx), as a proxy for excitatory neurotransmission and metabolism, to evaluate whether this negative result extended beyond the inhibitory neurotransmitter. There were no significant effects of viewing condition on Glx either (Fig. 4b, LMM, *F*_3,74.6_ = 0.97, *p* = 0.41). We also did not find any significant effects of ‘subtype’ on metabolite levels for GABA+ (*p* = 0.38) or Glx (*p* = 0.37). We found a significant effect of ‘age’ on metabolite levels (GABA+, *p* = 0.04), hence ‘age’ was controlled for in the main regression analysis of visual acuity loss with GABA+. Since there was no effect of viewing condition on metabolite concentrations across conditions, data were averaged across conditions to create a single GABA+ or Glx measure per person.

### 3.4 Weak evidence for a relationship between amblyopic visual acuity loss and visual cortex GABA+

**Fig. 5.**
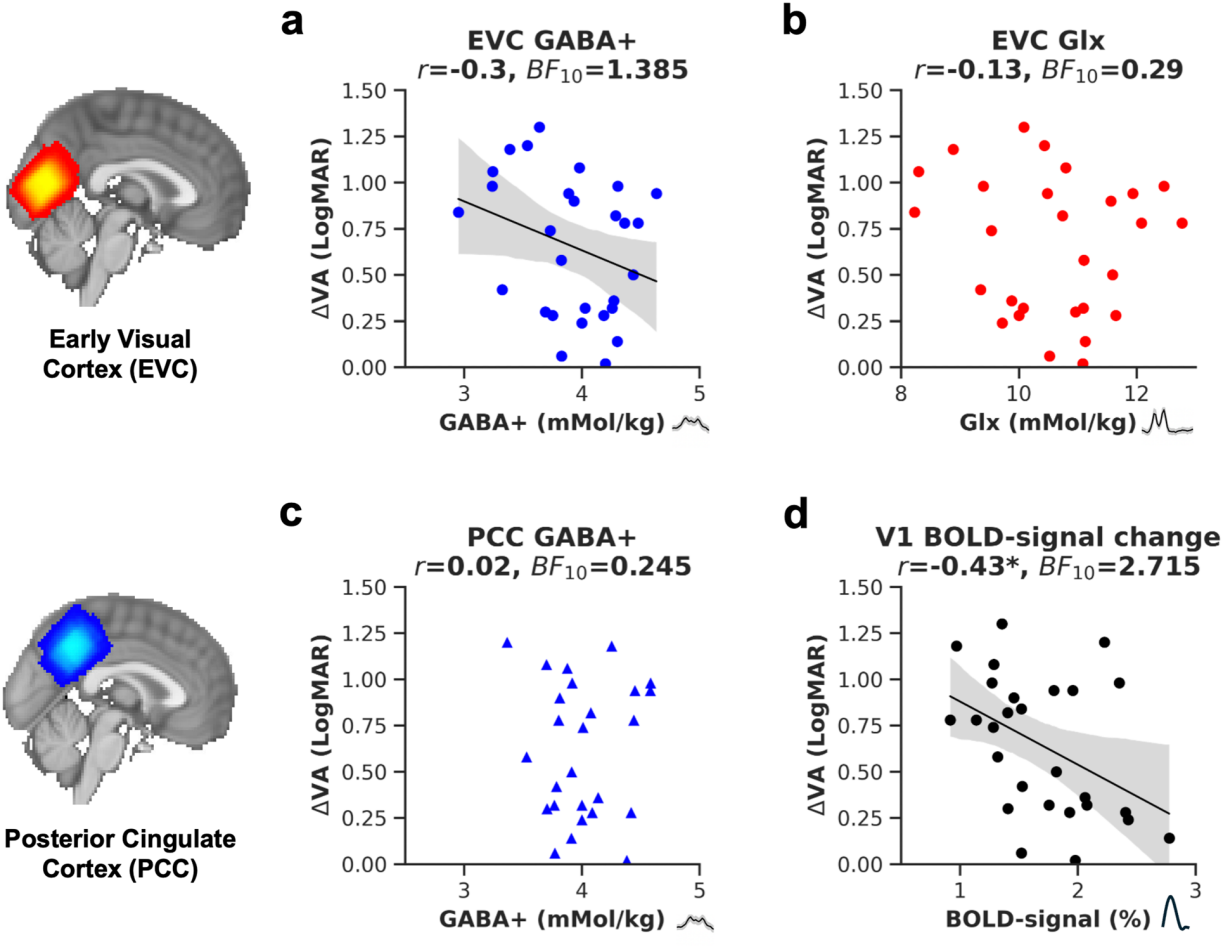
The relationship between visual acuity (VA) loss and GABA+, Glx and BOLD-signal change in the early visual cortex. Visual acuity loss is represented by subtracting the LogMAR visual acuity of the amblyopic from the fellow eye (ΔVA). The plot shows ΔVA plotted against GABA+ (a), or against Glx (b) in the early visual cortex (EVC). Additionally, we investigated the relationship between visual acuity loss and GABA+ in the posterior cingulate cortex (c) and hemodynamic changes in the primary visual cortex (V1) to flashing checkerboard stimuli (d). *r* = Pearson’s correlation coefficient, * = < 0.05 uncorrected *p* value, *BF*_10_ = Bayes Factor. The linear regression fit and 95% confidence interval of the linear regression line to the data were plotted where the *p* value was <0.1. Figure insets show the position of the MRS voxel.

A previous study with 14 participants found a strong negative correlation between visual acuity deficits and GABA+ (Mukerji et al., 2022). We sought to replicate this relationship in a larger sample. We found weak evidence for a negative relationship between ΔVA and EVC GABA+ (Fig. 5a, one-tailed Pearson’s correlation, *r* = -0.3, uncorrected *p* = 0.060, Bonferroni-adjusted *p* = 0.165, *BF*_10_ = 1.385). Controlling for age reduced the correlation (*r* = -0.22, uncorrected *p* = 0.138), suggesting that age contributed to the negative association. We also related GABA+/tCr to ΔVA. The measure did not show a strong negative correlation (GABA+/tCr, *r = *-0.16, uncorrected *p = *0.202, *BF*_10_ = 0.516). No relationship with ΔVA was found for Glx (Fig. 5b, Pearson’s correlation, *r* = -0.13, uncorrected *p* = 0.505, Bonferroni-adjusted *p* = 1, *BF*_10_ = 0.29), or for GABA+ from the PCC voxel (Fig. 5c, one-tailed Pearson’s correlation, n = 26, *r* = -0.02, uncorrected *p* = 0.916, Bonferroni-adjusted *p* = 1, *BF*_10_ = 0.245). None of the correlations were statistically significant after correcting for multiple comparisons.

We also explored the relationship between amblyopic visual acuity loss and hemodynamic responses to checkerboard stimuli in the primary visual cortex. Data were obtained from the fMRI localizer experiment, and the responses were quantified within a bilateral probabilistic V1 mask from the Jülich histological atlas (Amunts et al., 2020). Figure 5d shows that visual acuity loss correlated negatively with %BOLD-signal in V1 (*r = *-0.43, *p = *0.024, *BF*_10_ = 2.715). In other words, people with greater visual acuity loss due to amblyopia had lower visually-driven hemodynamic responses in V1.

**Fig. 6.**
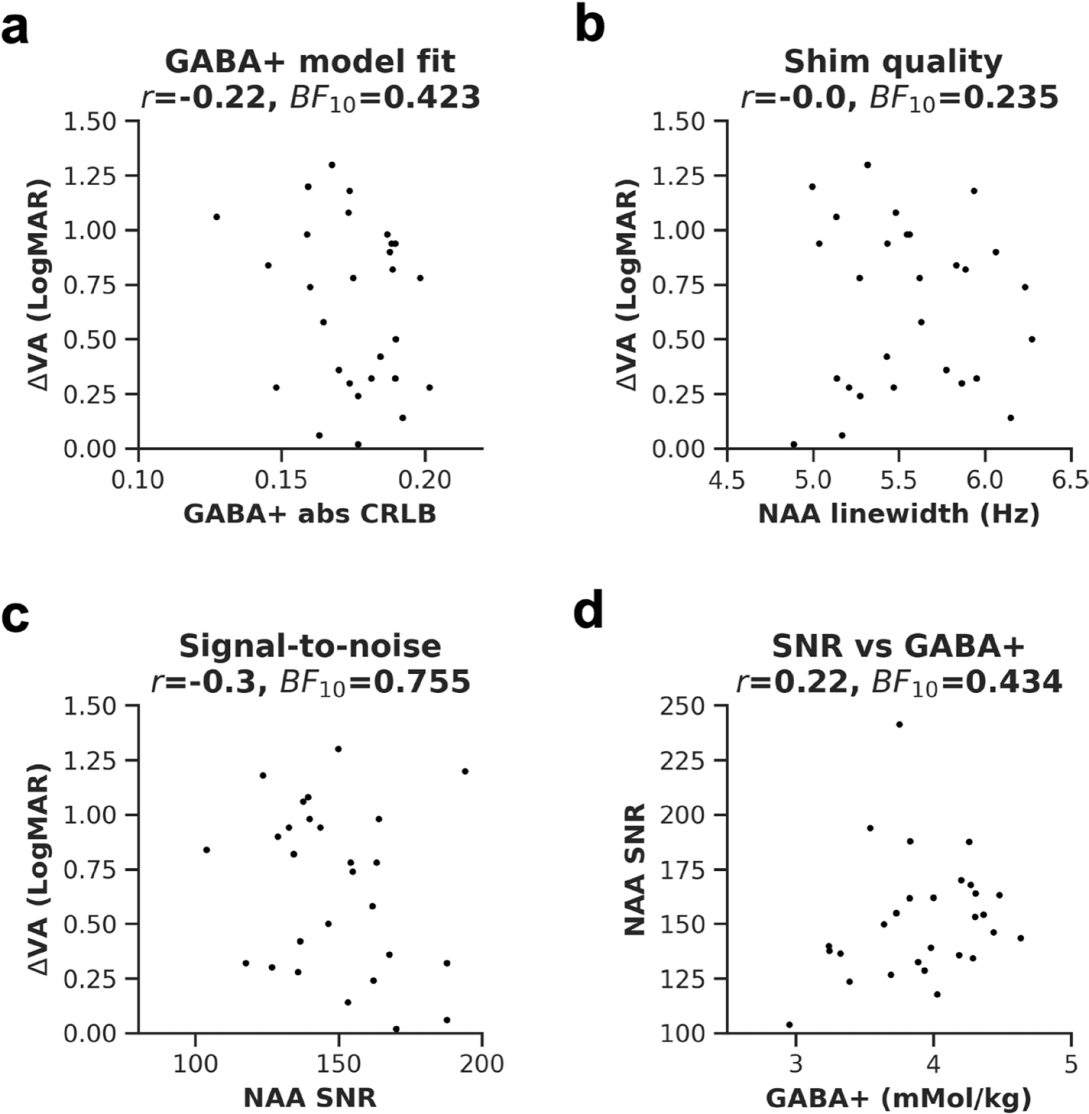
Data quality measures from the early visual cortex. GABA+ model fit (a), shim quality (b), and NAA SNR (c) were correlated with visual acuity differences. Also plotted is the correlation between SNR and GABA+ concentrations (d). EVC = early visual cortex. *r* = Pearson’s correlation coefficient, *BF*_10_ = Bayes Factor.

To evaluate the influence of data quality on the results, we assessed the relationship between visual acuity loss and three common MRS quality measures. We found no relationship with quality of GABA+ model fit (Fig. 6a, abs CRLB, *r* = -0.22, uncorrected *p* = 0.27, *BF*_10_ = 0.423), shim quality (Fig. 6b, NAA FWHM, *r* = 0.0, uncorrected *p* = 0.988, *BF*_10_ = 0.235), and signal-to-noise ratio (Fig. 6c, NAA SNR, *r = *-0.30, *p = *0.117, *BF*_10_ = 0.755). We also assessed the relationship between NAA SNR and GABA+ and found none (Fig. 6d, *r = *0.22, *p = *0.256, *BF*_10_ = 0.434).

Overall, these results show that the association between amblyopic visual acuity loss and visual cortex GABA+ is weak and influenced by participant age. The absence of a strong correlation was not due to the metabolite quantification method and was unlikely to have been directly influenced by MRS quality.

### 3.6 The association between visual acuity loss and GABA+ within amblyopia subtypes

**Fig. 7.**
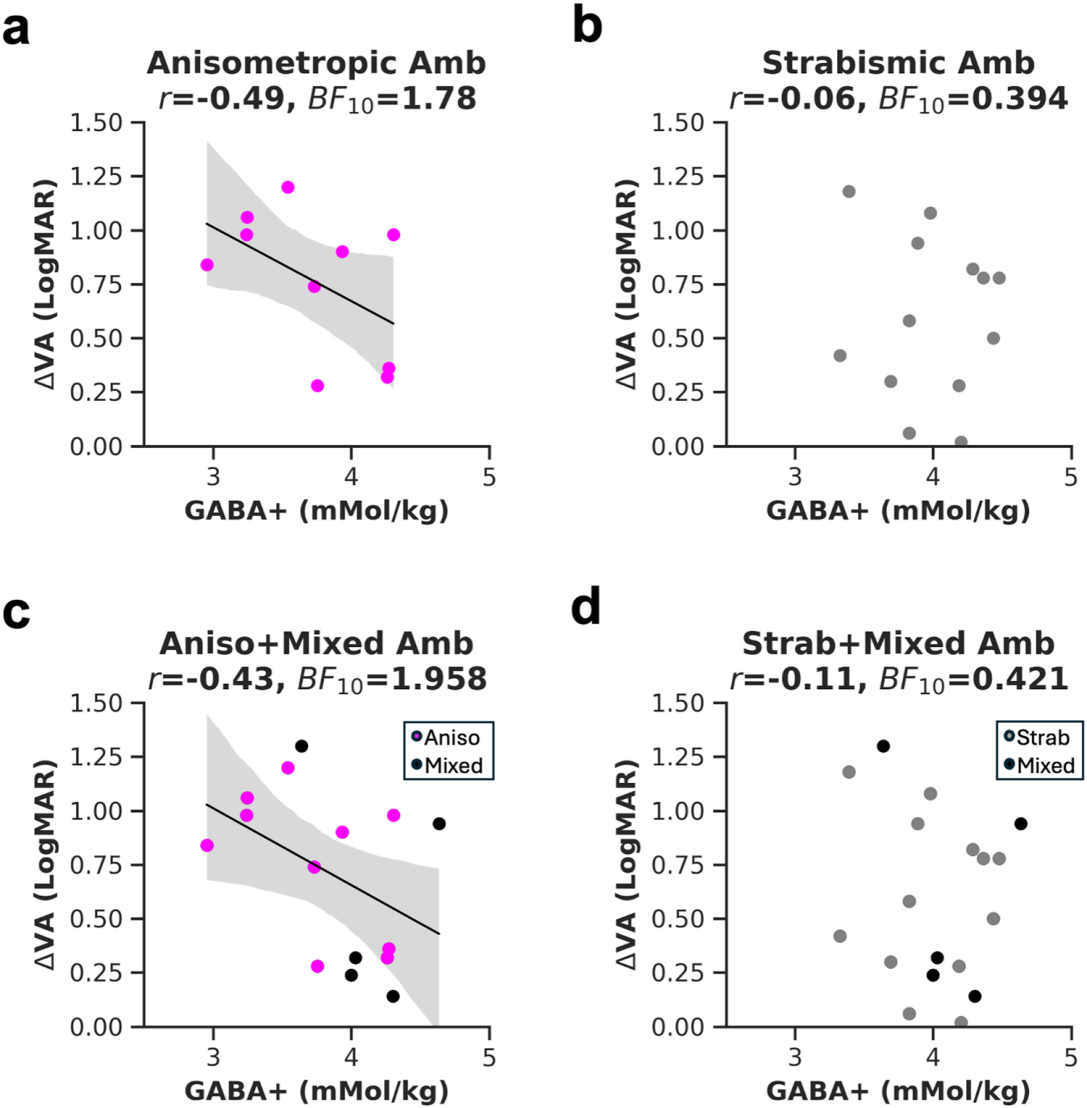
The relationship between visual acuity (VA) and GABA+ concentration in amblyopia subtypes. Visual acuity is represented by subtracting the LogMAR visual acuity of the fellow from the amblyopic eye (ΔVA). Correlation of ΔVA with EVC GABA+ in anisometropic amblyopes (a) or strabismic amblyopes (b). Mixed amblyopes (black dots) were grouped either with anisometropic (c), or with strabismic amblyopes (d). *r* = Pearson’s correlation coefficient, * = < 0.05 uncorrected *p* value, *BF*_10_ = Bayes Factor. The linear regression fit and 95% confidence interval of the linear regression line to the data were plotted where the *p* value was < 0.1.

Our study included amblyopes of different subtypes. Ten had anisometropia, 13 were strabismic, and five had mixed anisometropia and strabismus. In the following section, we characterize trends within amblyopia subtype using exploratory analyses. When characterizing the association by subtype, we found weak evidence for a negative relationship between ΔVA and EVC GABA+ for 10 anisometropic amblyopes (Fig. 7a: one-tailed Pearson’s correlation, *r = *-0.49, *p* = 0.074, *BF*_10_ = 1.78) and weak evidence for no association in 13 strabismic amblyopes (Fig. 7b: one-tailed Pearson’s correlation, *r = *-0.06, *p* = 0.43, *BF*_10_ = 0.394). A Fisher’s *r*-to-*z* transformation showed no significant difference between the two correlation coefficients (*z* = -0.97, *p* = 0.332), indicating that the two subgroups could not be dissociated from each other. Because mixed amblyopes can be grouped with either anisometropic or strabismic amblyopes, we pooled them with each group separately. We found weak evidence for a negative association for aniso+mixed amblyopes (Fig. 7c, one-tailed Pearson’s correlation for n = 15, *r = *-0.45, *p = *0.054, *BF*_10_ = 1.958), broadly consistent with a prior study (Mukerji et al., 2022) but with reduced correlation strength. When mixed amblyopes were grouped with strabismic amblyopes, we found again weak evidence for the null hypothesis (Fig. 7d, one-tailed Pearson’s correlation for n = 18, *r = *-0.11, *p = *0.33, *BF*_10_ = 0.421). The correlation coefficients between the two analyses did not differ (*z* = -0.97, *p* = 0.33). No evidence supporting the alternative hypothesis was found for the PCC voxel, irrespective of the grouping.

In summary, this section characterized the association between visual acuity deficits and GABA+ within amblyopia subtypes. Results show a difference in the strength of the relationship which was not statistically significant. These results suggest that the association between vision and neurochemistry may be influenced by the type of amblyopia.

## 4 Discussion

### 4.1 Summary

We evaluated the relationship between amblyopia and GABAergic inhibition in the adult human visual cortex. Our paradigm targeted the early visual cortex, where inputs from each eye arrive and are combined for binocular vision. Interocular suppression by the strong eye at this early stage is thought to drive visual abnormalities in amblyopia. Our study included the most common types of amblyopia, anisometropic and strabismic amblyopia. Contrary to our expectation, our paradigm did not reveal any interocular suppression via GABA+ in the visual cortex. When we related visual acuity deficits to GABA+, we found a weak negative association. In summary, our study, which includes the largest cohort of amblyopes in an MRS study to our knowledge, provides limited evidence for a relationship between GABAergic inhibition and visual acuity loss in human amblyopia.

### 4.2 The weak negative association between GABA+ with amblyopic visual acuity loss

We have previously shown in a small number of people (n = 14) with normal vision in both eyes, that greater eye dominance relates to lower GABA levels in the early visual cortex (Ip et al., 2021). Unlike normally sighted participants, amblyopes see primarily with their strong eye. Here, we find weak evidence for a negative association between GABA+ in the early visual cortex and visual acuity difference in amblyopes, with deeper amblyopia relating to lower GABA levels in the early visual cortex. The direction of the association suggests that failure of the amblyopic eye to inhibit the fellow eye disturbs the balance between eyes. This finding is consistent with psychophysical evidence showing that suppression from the amblyopic eye is abnormally weak, whereas inhibition from the fellow eye is comparable to normally sighted (Gong et al., 2020; Zhou et al., 2018). While we replicated the general direction of the negative association reported by a prior study (Mukerji et al., 2022), our correlation was not as strong. It is possible that the quantification method for the metabolites could explain the difference.

Our study reported GABA+ in absolute concentrations (Jansen et al., 2006), corrected for tissue-fraction while Mukerji et al. reported GABA+ relative to total creatine. Total creatine (creatine and phosphocreatine) is a widely used internal reference (de Graaf, 2007) which assumes that tCr is stable (Jansen et al., 2006). While this holds true in many cases, it also introduces ambiguity, as individual variability can either be driven by the metabolite or by tCr (Buonocore & Maddock, 2015; Li et al., 2003). In addition, while tCr provides an internal control for experimental conditions, it does not account for voxel tissue fraction. Controlling for tissue-composition is important (Harris et al., 2015), as GABA is known to be more abundant in grey than in white matter (Choi et al., 2006). Indeed, 3T measured GABA+ positively correlates with grey matter fraction (Craven et al., 2022). Without tissue-fraction correction, grey matter fraction can influence GABA+ estimation. However, our control analysis using GABA+/tCr suggests that no strong association is present irrespective of the metabolite quantification method. It is unlikely that the metabolite quantification method explains the difference between the two studies.

Our cohorts differed in size and in composition. With 28 participants, we had double the sample size of their study. While this increased our statistical power, our cohort also included strabismic amblyopes. Anisometropia and strabismus are the leading causes of amblyopia, each accounting for roughly 40% of cases reported (Harrington et al., 2019). Several studies provide information on differences between anisometropic and strabismic amblyopes or even directly compared them (Kiorpes et al., 1998; McKee et al., 2003; Wang et al., 2023). Strabismus may involve loss of long-range excitatory connections in the early visual cortex (Sengpiel & Blakemore, 1996). Supporting a difference, a recent SSVEP study found that while the response of anisometropic amblyopes to dichoptically presented gratings was comparable to controls, strabismic amblyopes had reduced responses, indicating reduced binocular interactions (Hou et al., 2021). It is possible that the strabismic amblyopes weakened the association in the present study. Indeed, we found that GABA+ in strabismic amblyopes was not associated with visual acuity deficits. Hence, it is possible that including participants with strabismic amblyopia accounted for the discrepancy between our and the previous study (Mukerji et al., 2022). While the finding was unexpected, the possibility of a subtype specific association with GABA merits further investigation.

### 4.3 No effects of viewing condition on GABA+ and Glx

Amblyopes grow up with unequal vision, viewing the world through their strong eye while their amblyopic eye is suppressed. We tested whether presenting monocular and binocular viewing conditions could reveal this powerful intracortical suppression, manifested as viewing-dependent differences in GABA+. Contrary to our expectations, we found no difference in GABA+ between viewing conditions. We thus replicated the result by Mukerji et al. (2022), extending it by including a larger number of participants and by investigating Glx concentrations. Our results that show no change in GABA+ when comparing stimulated to the rest condition in amblyopes also agree with studies in normally sighted (Bednarik et al., 2015; Mangia et al., 2007). However, our results are inconsistent with studies showing that GABA decreases with functional stimulation (Pasanta et al., 2023) and with time (Rideaux, 2020). It is possible that more perceptual conflict, or ongoing visual plasticity, is required to reveal viewing dependent GABAergic inhibition, a possibility that future studies can explore.

The lack of any effect of visual stimulation on Glx was surprising, and inconsistent with studies showing that glutamate and Glx increases with visual stimulation (Pasanta et al., 2023). A critical difference between our and previous studies was that we averaged across stimulus on and off periods within the same viewing condition, whereas other studies contrasted on and off periods within the same viewing condition. Using a comparable paradigm, however, Kurcyus et al. found significant increases in Glx/tCr between eyes closed and visually stimulated conditions (Kurcyus et al., 2018). Different amounts of visual attention may explain our negative and their positive results. The present study used 120 s blocks of on and off within condition while participants performed a fixation task, whereas Kurcyus et al. used 30 s blocks and subjects were instructed to pay attention to the stimulus or the fixation cross. It is possible that visual attention directed to the full-field checkerboards increased excitation in the visual stimulation conditions, as a previous study using PRESS at 3T has found that directed attention can modulate cortical Glx/tCr levels (Frank et al., 2021). In addition, using ‘eyes closed’ as comparison may have paradoxically increased Glx in the baseline condition. A prior study found that prolonged darkness increased visual cortex Glx/tCr levels in 16 participants using MEGA-PRESS at 3T (Min et al., 2023), and another study using a large cohort found a slow but steady rise in Glx/tCr over the acquisition time period, also using MEGA-PRESS at 3T (Rideaux, 2020). Future analysis of metabolite levels within stimulus on and off blocks may reveal if there are any changes in Glx within viewing conditions. Until then, the lack of any effect of visual stimulation on Glx adds to the heterogeneity of functional MRS findings (Pasanta et al., 2023).

### 4.4 The relationship between amblyopic visual acuity loss and the BOLD-signal in the primary visual cortex

Visual acuity loss in amblyopes was negatively associated with the %BOLD-change in the primary visual cortex during binocular viewing of flashing checkerboards in 27 adults with amblyopia. This means that participants with greater visual acuity loss had lower responses to visual stimuli. This finding is consistent with previous work comparing the amblyopic visual cortex hemodynamic response to that of normally sighted control participants (Baker et al., 2007; Clavagnier et al., 2015; Conner et al., 2007; Farivar et al., 2011; Goodyear et al., 2000; Hess et al., 2010; Lygo et al., 2021). Our preliminary results suggest that the hemodynamic response in the primary visual cortex during visual stimulation scales with visual acuity loss.

### 4.5 Limitations

We measured GABA+ from a control voxel in the posterior cingulate cortex. This allowed us to assess the regional specificity of our findings to the early visual cortex. Our quality control analysis showed that data quality differed between voxel locations: NAA signal-to-noise was better in the EVC, but shim quality was better in the PCC. MRS quality is known to vary between voxel locations, both in metabolite SNR and linewidth (Rideaux, 2020; Sanaei Nezhad et al., 2020). This means that neurochemistry measured from different locations may not be directly comparable.

The organization of the early visual cortex is such that the foveal representation is at the occipital pole. The visual cortex voxel was therefore placed as posterior as possible to optimize the inclusion of the foveal representation. Nonetheless, given the volume of the EVC voxel, it included more peripheral than central representation of V1. Similar EVC voxel position and size have been used to demonstrate associations between GABAergic inhibition in the early visual cortex and binocular rivalry dynamics in normally sighted participants (Ip et al., 2021; Lunghi et al., 2015; Robertson et al., 2016; van Loon et al., 2013) and visual acuity in amblyopes (Mukerji et al., 2022). The overrepresentation of the periphery would have been problematic if amblyopia affected only central vision, but this is not the case. Amblyopic impairments are more pronounced in the center but extend throughout the periphery. Peripheral deficits have been shown in visual field thresholds (Donahue et al., 1999; Greenstein et al., 2008), grating acuity (Mioche & Perenin, 1986), contrast sensitivity (Katz et al., 1984), spatial precision (Hussain & McGraw, 2022), stereovision (Verghese, 2023), and, importantly, interocular suppression (Babu et al., 2013, 2017; Sireteanu et al., 1981; Wiecek et al., 2024). Hence, central and peripheral V1 are meaningful to this study. More central V1 placements can be achieved with smaller voxels that fit into the occipital pole; this would come at the expense of acquisition time to maintain signal-to-noise comparable to prior studies. Greater overlap with central vision may evidence a stronger association between GABA and visual acuity differences.

Our study focuses on interocular visual acuity difference, the primary diagnostic measure for amblyopia in the clinic. We did not include a normally sighted cohort for the study, so we cannot comment on any group-level differences in GABAergic signaling between those whose vision developed normally and those who grew up amblyopic. However, normally sighted participants would have had normal visual acuity in both eyes, leading to minimal variability in the primary outcome measure of interocular difference in visual acuity. Other behavioral measures have been used to study visual impairments in amblyopia, and their relationship to GABAergic inhibition is largely unknown. Measures like binocular combination (Ding et al., 2013; Huang et al., 2009), dichoptic noise masking (Liu & Zhang, 2018, 2019), and residual stereopsis (Verghese, 2023) have been used previously to characterize amblyopic vision and could also provide a continuous measure within a control population.

## 5 Conclusion

In conclusion, this is the first study to examine the relationship between visual acuity loss and adult amblyopia in a cohort composed of the main three types of amblyopic subtypes: anisometropic, strabismic, and mixed amblyopia. Contrary to our expectations, we found only weak evidence for a negative association between visual cortex GABA+ and depth of amblyopia, as measured by the difference in visual acuity between the fellow and amblyopic eye in our cohort of 28 amblyopes. Our preliminary findings suggest that the type of amblyopia can influence the association, meaning that different composition of cohorts may reflect differential relationships between vision and the brain. Future studies with a greater number of participants in each subgroup can establish if the relationship to GABA is dissociable by the etiology of amblyopia.

## Data and Code Availability

The data for MRI imaging are available at https://zenodo.org/records/10425329. The MRS analysis code is available as part of the FSL-MRS distribution (version 2.1.19) at https://git.fmrib.ox.ac.uk/fsl/fsl_mrs/. MRS basis set is available at https://git.fmrib.ox.ac.uk/wclarke/win-mrs-basis-sets. Orthoptic data in Table 1 are available from https://git.fmrib.ox.ac.uk/betinaip/fmrs-amblyopia

## Author Contributions

I.B.I.: Conceptualization, Methodology, Formal analysis, Investigation, Writing—Original Draft, Writing—Review & Editing, Visualization, Supervision, Project administration, and Funding acquisition; W.T.C.: Methodology, Software, Resources, and Writing—Review & Editing; A.W.: Project administration, Resources, and Writing—Review & Editing; K.T.: Methodology, Writing—Review & Editing; J.M.: Methodology, Writing—Review & Editing; S.J.: Methodology, Writing—Review & Editing; L.S.: Resources, Writing—Review & Editing; S.T.: Resources, Writing—Review & Editing; H.W.: Resources, Writing—Review & Editing; L.B.: Investigation, Resources, and Writing—Review & Editing; A.J.P.: Conceptualization, Supervision, Project administration, Funding acquisition, and Writing—Review & Editing; H.B.: Conceptualization, Supervision, Project administration, Funding acquisition, and Writing—Review & Editing.

## Declaration Of Competing Interests

None.

## Supplementary Material

Supplementary Material

## References

[b1] Amunts, K., Malikovic, A., Mohlberg, H., Schormann, T., & Zilles, K. (2000). Brodmann’s areas 17 and 18 brought into stereotaxic space-where and how variable? Neuroimage, 11(1), 66–84. 10.1006/nimg.1999.0516 10686118

[b2] Amunts, K., Mohlberg, H., Bludau, S., & Zilles, K. (2020). Julich-Brain: A 3D probabilistic atlas of the human brain’s cytoarchitecture. Science, 369(6506), 988–992. 10.1126/science.abb4588 32732281

[b3] Babu, R. J., Clavagnier, S., Bobier, W. R., Thompson, B., & Hess, R. F. (2017). Regional extent of peripheral suppression in amblyopia. Invest Ophthalmol Vis Sci, 58(4), 2329–2340. 10.1167/iovs.16-20012 28431435

[b4] Babu, R. J., Clavagnier, S. R., Bobier, W., Thompson, B., & Hess, R. F. (2013). The regional extent of suppression: Strabismics versus nonstrabismics. Invest Ophthalmol Vis Sci, 54(10), 6585–6593. 10.1167/iovs.12-11314 24030466

[b5] Baker, D. H., Meese, T. S., Mansouri, B., & Hess, R. F. (2007). Binocular summation of contrast remains intact in strabismic amblyopia. Invest Ophthalmol Vis Sci, 48(11), 5332–5338. 10.1167/iovs.07-0194 17962490

[b6] Barnes, G. R., Hess, R. F., Dumoulin, S. O., Achtman, R. L., & Pike, G. B. (2001). The cortical deficit in humans with strabismic amblyopia. J Physiol, 533(Pt 1), 281–297. 10.1111/j.1469-7793.2001.0281b.x 11351035 PMC2278601

[b7] Bednarik, P., Tkac, I., Giove, F., DiNuzzo, M., Deelchand, D. K., Emir, U. E., Eberly, L. E., & Mangia, S. (2015). Neurochemical and BOLD responses during neuronal activation measured in the human visual cortex at 7 Tesla. J Cereb Blood Flow Metab, 35(4), 601–610. 10.1038/jcbfm.2014.233 25564236 PMC4420878

[b8] Birch, E. E. (2013). Amblyopia and binocular vision. Prog Retin Eye Res, 33, 67–84. 10.1016/j.preteyeres.2012.11.001 23201436 PMC3577063

[b9] Buonocore, M. H., & Maddock, R. J. (2015). Magnetic resonance spectroscopy of the brain: A review of physical principles and technical methods. Rev Neurosci, 26(6), 609–632. 10.1515/revneuro-2015-0010 26200810

[b10] Burchfield, J. L., & Duffy, F. H. (1981). Role of intracortical inhibition in deprivation amblyopia: Reversal by microiontophoretic bicuculline. Brain Res, 206(2), 479–484. 10.1016/0006-8993(81)90551-5 7214147

[b11] Button, K. S., Ioannidis, J. P., Mokrysz, C., Nosek, B. A., Flint, J., Robinson, E. S., & Munafo, M. R. (2013). Power failure: Why small sample size undermines the reliability of neuroscience. Nat Rev Neurosci, 14(5), 365–376. 10.1038/nrn3475 23571845

[b12] Choi, I. Y., Lee, S. P., Merkle, H., & Shen, J. (2006). In vivo detection of gray and white matter differences in GABA concentration in the human brain. Neuroimage, 33(1), 85–93. 10.1016/j.neuroimage.2006.06.016 16884929

[b13] Clarke, W. T., Bell, T. K., Emir, U. E., Mikkelsen, M., Oeltzschner, G., Shamaei, A., Soher, B. J., & Wilson, M. (2022). NIfTI-MRS: A standard data format for magnetic resonance spectroscopy. Magn Reson Med, 88(6), 2358–2370. 10.1002/mrm.29418 36089825 PMC7613677

[b14] Clarke, W. T., Stagg, C. J., & Jbabdi, S. (2021). FSL-MRS: An end-to-end spectroscopy analysis package. Magn Reson Med, 85(6), 2950–2964. 10.1002/mrm.28630 33280161 PMC7116822

[b15] Clavagnier, S., Dumoulin, S. O., & Hess, R. F. (2015). Is the cortical deficit in amblyopia due to reduced cortical magnification, loss of neural resolution, or neural disorganization? J Neurosci, 35(44), 14740–14755. 10.1523/JNEUROSCI.1101-15.2015 26538646 PMC6605231

[b16] Conner, I. P., Odom, J. V., Schwartz, T. L., & Mendola, J. D. (2007). Monocular activation of V1 and V2 in amblyopic adults measured with functional magnetic resonance imaging. J AAPOS, 11(4), 341–350. 10.1016/j.jaapos.2007.01.119 17434776 PMC2174609

[b17] Craven, A. R., Bhattacharyya, P. K., Clarke, W. T., Dydak, U., Edden, R. A. E., Ersland, L., Mandal, P. K., Mikkelsen, M., Murdoch, J. B., Near, J., Rideaux, R., Shukla, D., Wang, M., Wilson, M., Zollner, H. J., Hugdahl, K., & Oeltzschner, G. (2022). Comparison of seven modelling algorithms for gamma-aminobutyric acid-edited proton magnetic resonance spectroscopy. NMR Biomed, 35(7), e4702. 10.1002/nbm.4702 35078266 PMC9203918

[b18] de Graaf, R. A. (2007). In vivo NMR spectroscopy (2nd ed.). John Wiley & Sons, Ltd. 10.1002/9780470512968

[b19] Ding, J., Klein, S. A., & Levi, D. M. (2013). Binocular combination in abnormal binocular vision. J Vis, 13(2), 14. 10.1167/13.2.14 PMC4521338 23397039

[b20] Donahue, S. P., Wall, M., Kutzko, K. E., & Kardon, R. H. (1999). Automated perimetry in amblyopia: A generalized depression. Am J Ophthalmol, 127(3), 312–321. 10.1016/s0002-9394(98)90327-0 10088742

[b21] Farivar, R., Thompson, B., Mansouri, B., & Hess, R. F. (2011). Interocular suppression in strabismic amblyopia results in an attenuated and delayed hemodynamic response function in early visual cortex. J Vis, 11(14). 10.1167/11.14.16 22186274

[b22] Frank, S. M., Forster, L., Pawellek, M., Malloni, W. M., Ahn, S., Tse, P. U., & Greenlee, M. W. (2021). Visual attention modulates glutamate-glutamine levels in vestibular cortex: Evidence from magnetic resonance spectroscopy. J Neurosci, 41(9), 1970–1981. 10.1523/JNEUROSCI.2018-20.2020 33452222 PMC7939083

[b23] Fu, Z., Hong, H., Su, Z., Lou, B., Pan, C. W., & Liu, H. (2020). Global prevalence of amblyopia and disease burden projections through 2040: A systematic review and meta-analysis. Br J Ophthalmol, 104(8), 1164–1170. 10.1136/bjophthalmol-2019-314759 31704700

[b24] Gong, L., Reynaud, A., Wang, Z., Cao, S., Lu, F., Qu, J., Hess, R. F., & Zhou, J. (2020). Interocular suppression as revealed by dichoptic masking is orientation-dependent and imbalanced in amblyopia. Invest Ophthalmol Vis Sci, 61(14), 28. 10.1167/iovs.61.14.28 PMC7774058 33369637

[b25] Goodyear, B. G., Nicolle, D. A., Humphrey, G. K., & Menon, R. S. (2000). BOLD fMRI response of early visual areas to perceived contrast in human amblyopia. J Neurophysiol, 84(4), 1907–1913. 10.1152/jn.2000.84.4.1907 11024083

[b26] Greenstein, V. C., Eggers, H. M., & Hood, D. C. (2008). Multifocal visual evoked potential and automated perimetry abnormalities in strabismic amblyopes. J AAPOS, 12(1), 11–17. 10.1016/j.jaapos.2007.04.017 17651996 PMC2359226

[b27] Grieco, S. F., Qiao, X., Zheng, X., Liu, Y., Chen, L., Zhang, H., Yu, Z., Gavornik, J. P., Lai, C., Gandhi, S. P., Holmes, T. C., & Xu, X. (2020). Subanesthetic ketamine reactivates adult cortical plasticity to restore vision from amblyopia. Curr Biol, 30(18), 3591–3603 e3598. 10.1016/j.cub.2020.07.008 32822611 PMC7925140

[b28] Hallum, L. E., Shooner, C., Kumbhani, R. D., Kelly, J. G., Garcia-Marin, V., Majaj, N. J., Movshon, J. A., & Kiorpes, L. (2017). Altered balance of receptive field excitation and suppression in visual cortex of amblyopic macaque monkeys. J Neurosci, 37(34), 8216–8226. 10.1523/JNEUROSCI.0449-17.2017 28743725 PMC5566869

[b29] Harrington, S., Breslin, K., O’Dwyer, V., & Saunders, K. (2019). Comparison of amblyopia in schoolchildren in Ireland and Northern Ireland: A population-based observational cross-sectional analysis of a treatable childhood visual deficit. BMJ Open, 9(8), e031066. 10.1136/bmjopen-2019-031066 PMC6701591 31401612

[b30] Harris, A. D., Puts, N. A., & Edden, R. A. (2015). Tissue correction for GABA-edited MRS: Considerations of voxel composition, tissue segmentation, and tissue relaxations. J Magn Reson Imaging, 42(5), 1431–1440. 10.1002/jmri.24903 26172043 PMC4615266

[b31] Hensch, T. K., & Quinlan, E. M. (2018). Critical periods in amblyopia. Vis Neurosci, 35, E014. 10.1017/S0952523817000219 29905116 PMC6047524

[b32] Hess, R. F., Li, X., Lu, G., Thompson, B., & Hansen, B. C. (2010). The contrast dependence of the cortical fMRI deficit in amblyopia; a selective loss at higher contrasts. Hum Brain Mapp, 31(8), 1233–1248. 10.1002/hbm.20931 20063352 PMC6870632

[b33] Hou, C., Tyson, T. L., Uner, I. J., Nicholas, S. C., & Verghese, P. (2021). Excitatory contribution to binocular interactions in human visual cortex is reduced in strabismic amblyopia. Journal of Neuroscience, 41(41), 8632–8643. 10.1523/Jneurosci.0268-21.2021 34433631 PMC8513700

[b34] Hu, B., Liu, Z., Zhao, J., Zeng, L., Hao, G., Shui, D., & Mao, K. (2022). The global prevalence of amblyopia in children: A systematic review and meta-analysis. Front Pediatr, 10, 819998. 10.3389/fped.2022.819998 35601430 PMC9114436

[b35] Huang, C. B., Zhou, J., Lu, Z. L., Feng, L., & Zhou, Y. (2009). Binocular combination in anisometropic amblyopia. J Vis, 9(3), 17 11–16. 10.1167/9.3.17 19757956 PMC2861488

[b36] Hubel, D. H., & Wiesel, T. N. (1965). Binocular interaction in striate cortex of kittens reared with artificial squint. J Neurophysiol, 28(6), 1041–1059. 10.1152/jn.1965.28.6.1041 5883731

[b37] Hussain, Z., & McGraw, P. V. (2022). Disruption of positional encoding at small separations in the amblyopic periphery. Invest Ophthalmol Vis Sci, 63(4), 15. 10.1167/iovs.63.4.15 PMC9034712 35446345

[b38] Huttunen, H. J., Palva, J. M., Lindberg, L., Palva, S., Saarela, V., Karvonen, E., Latvala, M. L., Liinamaa, J., Booms, S., Castren, E., & Uusitalo, H. (2018). Fluoxetine does not enhance the effect of perceptual learning on visual function in adults with amblyopia. Sci Rep, 8(1), 12830. 10.1038/s41598-018-31169-z 30150750 PMC6110780

[b39] Ip, I. B., Emir, U. E., Lunghi, C., Parker, A. J., & Bridge, H. (2021). GABAergic inhibition in the human visual cortex relates to eye dominance. Sci Rep, 11(1), 17022. 10.1038/s41598-021-95685-1 34426611 PMC8382755

[b40] Jansen, J. F., Backes, W. H., Nicolay, K., & Kooi, M. E. (2006). 1H MR spectroscopy of the brain: Absolute quantification of metabolites. Radiology, 240(2), 318–332. 10.1148/radiol.2402050314 16864664

[b41] Jenkinson, M., Bannister, P., Brady, M., & Smith, S. (2002). Improved optimization for the robust and accurate linear registration and motion correction of brain images. Neuroimage, 17(2), 825–841. 10.1016/s1053-8119(02)91132-8 12377157

[b42] Jenkinson, M., & Smith, S. (2001). A global optimisation method for robust affine registration of brain images. Med Image Anal, 5(2), 143–156. 10.1016/s1361-8415(01)00036-6 11516708

[b43] Joly, O., & Franko, E. (2014). Neuroimaging of amblyopia and binocular vision: A review. Front Integr Neurosci, 8, 62. 10.3389/fnint.2014.00062 25147511 PMC4123726

[b44] Katz, L. M., Levi, D. M., & Bedell, H. E. (1984). Central and peripheral contrast sensitivity in amblyopia with varying field size. Doc Ophthalmol, 58(4), 351–373. 10.1007/BF00679799 6525936

[b45] Kiorpes, L. (2019). Understanding the development of amblyopia using macaque monkey models. Proc Natl Acad Sci U S A, 116(52), 26217–26223. 10.1073/pnas.1902285116 31871163 PMC6936699

[b46] Kiorpes, L., Kiper, D. C., O’Keefe, L. P., Cavanaugh, J. R., & Movshon, J. A. (1998). Neuronal correlates of amblyopia in the visual cortex of macaque monkeys with experimental strabismus and anisometropia. J Neurosci, 18(16), 6411–6424. 10.1523/JNEUROSCI.18-16-06411.1998 9698332 PMC6793177

[b47] Kurcyus, K., Annac, E., Hanning, N. M., Harris, A. D., Oeltzschner, G., Edden, R., & Riedl, V. (2018). Opposite dynamics of GABA and glutamate levels in the occipital cortex during visual processing. J Neurosci, 38(46), 9967–9976. 10.1523/JNEUROSCI.1214-18.2018 30282724 PMC6234295

[b48] Lagas, A. K., Black, J. M., Russell, B. R., Kydd, R. R., & Thompson, B. (2019). The effect of combined patching and citalopram on visual acuity in adults with amblyopia: A randomized, crossover, placebo-controlled trial. Neural Plast, 2019, 5857243. 10.1155/2019/5857243 31281343 PMC6590556

[b49] Lakens, D. (2022). Sample size justification. Collabra: Psychology, 8(1), 33267. 10.1525/collabra.33267

[b50] Li, B. S., Wang, H., & Gonen, O. (2003). Metabolite ratios to assumed stable creatine level may confound the quantification of proton brain MR spectroscopy. Magn Reson Imaging, 21(8), 923–928. 10.1016/s0730-725x(03)00181-4 14599543

[b51] Liu, X. Y., & Zhang, J. Y. (2018). Dichoptic training in adults with amblyopia: Additional stereoacuity gains over monocular training. Vision Res, 152, 84–90. 10.1016/j.visres.2017.07.002 28736224

[b52] Liu, X. Y., & Zhang, J. Y. (2019). Dichoptic de-masking learning in adults with amblyopia and its mechanisms. Invest Ophthalmol Vis Sci, 60(8), 2968–2977. 10.1167/iovs.18-26483 31307059

[b53] Lunghi, C., Emir, U. E., Morrone, M. C., & Bridge, H. (2015). Short-term monocular deprivation alters GABA in the adult human visual cortex. Curr Biol, 25(11), 1496–1501. 10.1016/j.cub.2015.04.021 26004760 PMC5040500

[b54] Lygo, F. A., Richard, B., Wade, A. R., Morland, A. B., & Baker, D. H. (2021). Neural markers of suppression in impaired binocular vision. Neuroimage, 230, 117780. 10.1016/j.neuroimage.2021.117780 33503479 PMC8063178

[b55] Mangia, S., Tkac, I., Gruetter, R., Van de Moortele, P. F., Maraviglia, B., & Ugurbil, K. (2007). Sustained neuronal activation raises oxidative metabolism to a new steady-state level: Evidence from 1 H NMR spectroscopy in the human visual cortex. J Cereb Blood Flow Metab, 27(5), 1055–1063. 10.1038/sj.jcbfm.9600401 17033694

[b56] Maya Vetencourt, F. J., Sale, A., Viegi, A., Baroncelli, L., De Pasquale, R., O’Leary, O. F., Castren, E., & Maffei, L. (2008). The antidepressant fluoxetine restores plasticity in the adult visual cortex. Science, 320(5874), 385–388. 10.1126/science.1150516 18420937

[b57] McKee, S. P., Levi, D. M., & Movshon, J. A. (2003). The pattern of visual deficits in amblyopia. J Vis, 3(5), 380–405. 10.1167/3.5.5 12875634

[b58] McKinney, W. (2010). Data structures for statistical computing in python. Proceedings of the 9th Python in Science Conference, 445, 51–56. 10.25080/Majora-92bf1922-00a

[b59] Mescher, M., Merkle, H., Kirsch, J., Garwood, M., & Gruetter, R. (1998). Simultaneous in vivo spectral editing and water suppression. NMR Biomed, 11(6), 266–272. doi: 10.1002/(sici)1099-1492(199810)11:6<266::aid-nbm530>3.0.co;2-j 9802468

[b60] Min, S. H., Wang, Z., Chen, M. T., Hu, R., Gong, L., He, Z., Wang, X., Hess, R. F., & Zhou, J. (2023). Metaplasticity: Dark exposure boosts local excitability and visual plasticity in adult human cortex. J Physiol, 601(18), 4105–4120. 10.1113/JP284040 37573529

[b61] Mioche, L., & Perenin, M. T. (1986). Central and peripheral residual vision in humans with bilateral deprivation amblyopia. Exp Brain Res, 62(2), 259–272. 10.1007/BF00238845 3709711

[b62] Mitchell, D., & Sengpiel, F. (2018). Animal models of amblyopia. Vis Neurosci, 35, E017. 10.1017/S0952523817000244 29905121

[b63] Mukerji, A., Byrne, K. N., Yang, E. N. C., Levi, D. M., & Silver, M. A. (2022). Visual cortical gamma-aminobutyric acid and perceptual suppression in amblyopia. Front Hum Neurosci, 16, 949395. 10.3389/fnhum.2022.949395 36118971 PMC9479630

[b64] Nuzzo, R. L. (2017). An introduction to Bayesian data analysis for correlations. PM R, 9(12), 1278–1282. 10.1016/j.pmrj.2017.11.003 29274678

[b65] Pan, Y., Tarczy-Hornoch, K., Cotter, S. A., Wen, G., Borchert, M. S., Azen, S. P., Varma, R., & Study Multi-Ethnic Pediatric Eye Disease, G. (2009). Visual acuity norms in pre-school children: The Multi-Ethnic Pediatric Eye Disease Study. Optom Vis Sci, 86(6), 607–612. 10.1097/OPX.0b013e3181a76e55 19430325 PMC2742505

[b66] Pasanta, D., He, J. L., Ford, T., Oeltzschner, G., Lythgoe, D. J., & Puts, N. A. (2023). Functional MRS studies of GABA and glutamate/Glx - A systematic review and meta-analysis. Neurosci Biobehav Rev, 144, 104940. 10.1016/j.neubiorev.2022.104940 36332780 PMC9846867

[b67] Pitchaimuthu, K., Wu, Q. Z., Carter, O., Nguyen, B. N., Ahn, S., Egan, G. F., & McKendrick, A. M. (2017). Occipital GABA levels in older adults and their relationship to visual perceptual suppression. Sci Rep, 7(1), 14231. 10.1038/s41598-017-14577-5 29079815 PMC5660206

[b68] Rideaux, R. (2020). Temporal dynamics of GABA and Glx in the visual cortex. eNeuro, 7(4). 10.1523/ENEURO.0082-20.2020 PMC7429906 32571964

[b69] Robertson, C. E., Ratai, E. M., & Kanwisher, N. (2016). Reduced GABAergic action in the autistic brain. Curr Biol, 26(1), 80–85. 10.1016/j.cub.2015.11.019 26711497

[b70] Rothman, D. L., Behar, K. L., Prichard, J. W., & Petroff, O. A. (1997). Homocarnosine and the measurement of neuronal pH in patients with epilepsy. Magn Reson Med, 38(6), 924–929. 10.1002/mrm.1910380611 9402193

[b71] Sanaei Nezhad, F., Lea-Carnall, C. A., Anton, A., Jung, J., Michou, E., Williams, S. R., & Parkes, L. M. (2020). Number of subjects required in common study designs for functional GABA magnetic resonance spectroscopy in the human brain at 3 Tesla. Eur J Neurosci, 51(8), 1784–1793. 10.1111/ejn.14618 31705723 PMC7216844

[b72] Sengpiel, F., & Blakemore, C. (1996). The neural basis of suppression and amblyopia in strabismus. Eye (Lond), 10(Pt 2), 250–258. 10.1038/eye.1996.54 8776456

[b73] Sengpiel, F., Jirmann, K. U., Vorobyov, V., & Eysel, U. T. (2006). Strabismic suppression is mediated by inhibitory interactions in the primary visual cortex. Cereb Cortex, 16(12), 1750–1758. 10.1093/cercor/bhj110 16400161

[b74] Sharif, M. H., Talebnejad, M. R., Rastegar, K., Khalili, M. R., & Nowroozzadeh, M. H. (2019). Oral fluoxetine in the management of amblyopic patients aged between 10 and 40 years old: A randomized clinical trial. Eye (Lond), 33(7), 1060–1067. 10.1038/s41433-019-0360-z 30783259 PMC6707246

[b75] Sireteanu, R., Fronius, M., & Singer, W. (1981). Binocular interaction in the peripheral visual field of humans with strabismic and anisometropic amblyopia. Vision Res, 21(7), 1065–1074. 10.1016/0042-6989(81)90011-0 7314487

[b76] Smith, S. M. (2002). Fast robust automated brain extraction. Hum Brain Mapp, 17(3), 143–155. 10.1002/hbm.10062 12391568 PMC6871816

[b77] Tiew, S., Lim, C., & Sivagnanasithiyar, T. (2020). Using an excel spreadsheet to convert Snellen visual acuity to LogMAR visual acuity. Eye (Lond), 34(11), 2148–2149. 10.1038/s41433-020-0783-6 32020059 PMC7784686

[b78] Vallat, R. (2018). Pingouin: Statistics in Python. J Open Source Softw, 3(31), 1026. 10.21105/joss.01026

[b79] van Loon, A. M., Knapen, T., Scholte, H. S., St John-Saaltink, E., Donner, T. H., & Lamme, V. A. (2013). GABA shapes the dynamics of bistable perception. Curr Biol, 23(9), 823–827. 10.1016/j.cub.2013.03.067 23602476

[b80] Verghese, P. (2023). The utility of peripheral stereopsis. Front Neurosci, 17, 1217993. 10.3389/fnins.2023.1217993 37795187 PMC10545962

[b81] Wang, Y., Wu, Y., Luo, L., & Li, F. (2023). Structural and functional alterations in the brains of patients with anisometropic and strabismic amblyopia: A systematic review of magnetic resonance imaging studies. Neural Regen Res, 18(11), 2348–2356. 10.4103/1673-5374.371349 37282452 PMC10360096

[b82] Wiecek, E., Kosovicheva, A., Ahmed, Z., Nabasaliza, A., Kazlas, M., Chan, K., Hunter, D. G., & Bex, P. J. (2024). Peripheral binocular imbalance in anisometropic and strabismic amblyopia. Invest Ophthalmol Vis Sci, 65(4), 36. 10.1167/iovs.65.4.36 PMC11044833 38652649

[b83] Wiesel, T. N., & Hubel, D. H. (1963). Single-cell responses in striate cortex of kittens deprived of vision in one eye. J Neurophysiol, 26, 1003–1017. 10.1152/jn.1963.26.6.1003 14084161

[b84] Worsley, K. J. (2001). Statistical analysis of activation images. In P. Jezzard, P. M. Matthews, & S. M. Smith (Eds.), Functional Magnetic Resonance Imaging: An Introduction to Methods. Oxford: Oxford Academic. 10.1093/acprof:oso/9780192630711.003.0014

[b85] Zhou, J., Reynaud, A., Yao, Z., Liu, R., Feng, L., Zhou, Y., & Hess, R. F. (2018). Amblyopic suppression: Passive attenuation, enhanced dichoptic masking by the fellow eye or reduced dichoptic masking by the amblyopic eye? Invest Ophthalmol Vis Sci, 59(10), 4190–4197. 10.1167/iovs.18-24206 30128490

[b86] Zollner, H. J., Oeltzschner, G., Schnitzler, A., & Wittsack, H. J. (2021). In silico GABA+ MEGA-PRESS: Effects of signal-to-noise ratio and linewidth on modeling the 3 ppm GABA+ resonance. NMR Biomed, 34(1), e4410. 10.1002/nbm.4410 32989890 PMC8935357

