# Supplementary material for: The relationship between visual acuity loss and GABAergic inhibition in amblyopia
Source: Imaging Neurosci (Camb). 2024 Aug 6;2:imag-2-00256. doi: 10.1162/imag_a_00256 (PMC11917722; doi:10.1162/imag_a_00256)
Supplement: Supplementary Material [file imag_a_00256-supp.pdf]

Supplementary Materials

Minimum Reporting Standards for MRS Checklist

Site (Name or Number)	1
1. Hardware	
a. Field strength [T]	3T
b. Manufacturer	Siemens
c. Model (software version if available)	Prisma (VE11E)
d. RF coils: nuclei (transmit/ receive), number of channels, type, body part	Siemens 64 channel head coil.
e. Additional hardware	N/A
2. Acquisition	
a. Pulse sequence	MEGA-PRESS<br><br>We used a locally developed MEGA-PRESS sequence, derived from the CMRR spectroscopy package MEGA-PRESS sequence (https://www.cmrr.umn.edu/spectro/).<br><br>1. Tremblay S, Beaulé V, Proulx S, Lafleur LP, Doyon J, Marjańska M, Théoret H. The use of magnetic resonance spectroscopy as a tool for the measurement of bi-hemispheric transcranial electric stimulation effects on primary motor cortex metabolism. J Vis Exp 2014;93,e51631.<br>2. Marjańska M, Lehéricy S, Valabrègue R, Popa T, Worbe Y, Russo M, Auerbach EJ, Grabli D, Bonnet C, Gallea C, Coudert M, Yahia-Cherif L, Vidailhet M, Meunier C. Brain dynamic neurochemical changes in dystonic patients: a magnetic resonance spectroscopy study. Mov Disord. 2013;28:201-9.

b. Volume of Interest (VOI) locations	Primary visual cortex voxel (EVC)<br><br>Posterior cingulate cortex (PCC)
c. Nominal VOI size [cm^3, mm^3]	Anterior - Posterior: 20 mm<br><br>Left - Right: 25 mm<br><br>Head - Foot: 25 mm
d. Repetition Time (TR), Echo Time (TE) [ms, s]	TR = 1500ms<br><br>TE = 68ms
e. Total number of Excitations or acquisitions per spectrum<br><br>In time series for kinetic studies<br><br>i. Number of Averaged spectra (NA) per time-point<br>ii. Averaging method (e.g. block-wise or moving average)<br>iii. Total number of spectra (acquired / in time-series)	160 edit-on and 160 edit-off per editing condition (320 total)
f. Additional sequence parameters<br><br>(spectral width in Hz, number of spectral	4000Hz<br><br>2048 points

points, frequency offsets)<br><br>If STEAM:, Mixing Time (TM)<br><br>If MRSI: 2D or 3D, FOV in all directions, matrix size, acceleration factors, sampling method	
g. Water Suppression Method	VAPOR with additional water suppression using dual-band editing pulse.
h. Shimming Method, reference peak, and thresholds for "acceptance of shim" chosen	Automated, vendor supplied 3D GRE B_0 field mapping technique ("GRE Brain") was used to ensure that vendor-reported full-width-at-half-maximum (FWHM) were below 20 Hz, and that water-unsuppressed MRS-measured FWHM were <12 Hz.
i. Triggering or motion correction method<br><br>(respiratory, peripheral, cardiac triggering, incl. device used and delays)	None
**3. Data analysis methods and outputs**	

a. Analysis software	FSL-MRS 2.1.19
b. Processing steps deviating from quoted reference or product	fsl_mrs_preproc_edit \ --data data.nii.gz \ # metabolite file --reference data_phasecorr.nii.gz \ # combined phase corrected water reference --output /preproc_data \ # output directory --leftshift 3 \ # preprocessing parameter --hlsvd \ # residual water removal --report \ --overwrite \ --align_window_dynamic 16 \ --align_ppm_edit 2.5 3.5 \ # phase/frequency alignment range
c. Output measure<br><br>(e.g. absolute concentration, institutional units, ratio) Processing steps deviating from quoted reference or product	Absolute concentrations (mMol/kg)
d. Quantification references and assumptions, fitting model assumptions	fsl_mrs \ --data diff.nii.gz \ --h2o wref.nii.gz \ # water reference file --tissue_frac tissue_fraction.json \ # tissue fraction file --basis uzay_svs_mpress_68_with_db/diff \ # basis set --metab_groups sysMM \ # grouping --keep GABA GSH Glu Gln NAA NAAG sysMM \. # fitting --combine Glu Gln GSH\ --combine GABA sysMM \ --combine NAA NAAG \

	--internal_ref NAA \ --baseline_order -1 \ --output /fit_output/data \ --overwrite \ --report \ <br>[1] Clarke WT, Stagg CJ, Jbabdi S. FSL-MRS: An end-to-end spectroscopy analysis package. Magnetic Resonance in Medicine 2021;85:2950-2964 doi: 10.1002/mrm.28630.
**4. Data Quality**	
a. Reported variables<br><br>(SNR, Linewidth (with reference peaks))	FSL-MRS reported linewidth of the inverted NAA peak (FWHM).<br><br>FSL-MRS reported NAA SNR of the NAA peak in the edit-off spectrum.
b. Data exclusion criteria	Univariate outliers: 1.5 interquartile range in metabolite concentrations.
c. Quality measures of postprocessing Model fitting (e.g. CRLB, goodness of fit, SD of residual)	FSL-MRS reported absolute Cramer-Rao lower bounds (CRLB).

d. Sample Spectrum	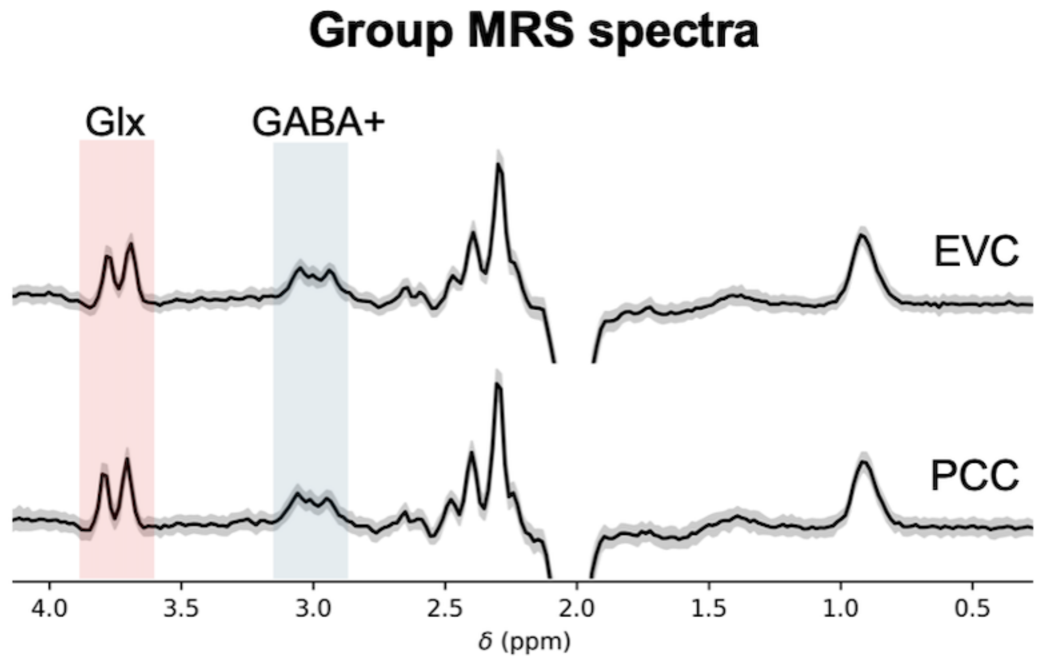
